# *Borrelia burgdorferi* BBK32 Inhibits the Classical Pathway by Blocking Activation of the C1 Complement Complex

**DOI:** 10.1371/journal.ppat.1005404

**Published:** 2016-01-25

**Authors:** Brandon L. Garcia, Hui Zhi, Beau Wager, Magnus Höök, Jon T. Skare

**Affiliations:** 1 Department of Biochemistry and Molecular Biophysics, Kansas State University, Manhattan, Kansas, United States of America; 2 Center for Infectious and Inflammatory Diseases, Institute of Biosciences and Technology, Texas A&M Health Science Center, Houston, Texas, United States of America; 3 Department of Microbial Pathogenesis and Immunology, College of Medicine, Texas A&M Health Science Center, Bryan, Texas, United States of America; University of Montana, UNITED STATES

## Abstract

Pathogens that traffic in blood, lymphatics, or interstitial fluids must adopt strategies to evade innate immune defenses, notably the complement system. Through recruitment of host regulators of complement to their surface, many pathogens are able to escape complement-mediated attack. The Lyme disease spirochete, *Borrelia burgdorferi*, produces a number of surface proteins that bind to factor H related molecules, which function as the dominant negative regulator of the alternative pathway of complement. Relatively less is known about how *B*. *burgdorferi* evades the classical pathway of complement despite the observation that some *sensu lato* strains are sensitive to classical pathway activation. Here we report that the borrelial lipoprotein BBK32 potently and specifically inhibits the classical pathway by binding with high affinity to the initiating C1 complex of complement. In addition, *B*. *burgdorferi* cells that produce BBK32 on their surface bind to both C1 and C1r and a serum sensitive derivative of *B*. *burgdorferi* is protected from killing via the classical pathway in a BBK32-dependent manner. Subsequent biochemical and biophysical approaches localized the anti-complement activity of BBK32 to its globular C-terminal domain. Mechanistic studies reveal that BBK32 acts by entrapping C1 in its zymogen form by binding and inhibiting the C1 subcomponent, C1r, which serves as the initiating serine protease of the classical pathway. To our knowledge this is the first report of a spirochetal protein acting as a direct inhibitor of the classical pathway and is the only example of a biomolecule capable of specifically and noncovalently inhibiting C1/C1r. By identifying a unique mode of complement evasion this study greatly enhances our understanding of how pathogens subvert and potentially manipulate host innate immune systems.

## Author Summary

The human complement system is a connected network of blood proteins capable of recognizing and eliminating microbial intruders. To avoid the destructive force of complement activation, many microorganisms that enter the bloodstream express molecules that disrupt key steps of the complement cascade by interacting with specific complement components. In this study we show that the causative agent of Lyme disease, *Borrelia burgdorferi*, expresses a surface-protein termed BBK32 that targets and inhibits the first component of complement, designated C1. Upon binding to human C1, BBK32 traps this initiating protease complex of the classical pathway of complement in an inactive state, and prevents the downstream proteolytic events of the pathway. Our study defines a new mechanism by which microbes are able to escape the human innate immune system and identifies complement protease C1r as a previously unknown target of bacterial anti-complement molecules. Thus, discovery of the complement inhibitory activity of the borrelial protein BBK32 significantly advances our understanding of how disease-causing bacteria survive in immune competent hosts.

## Introduction

Complement is an interconnected system of serum and cell surface proteins that comprises a primary arm of innate immunity. Complement acts in the recognition and clearance of microbial invaders and terminal activation of the complement cascade can result in direct lysis of pathogens. In addition to its function as a ‘first-line-of-defense’, complement plays an extensive role in mediating inflammatory responses, maintaining cell homeostasis, and in coordinating tissue development and repair [1]. Complement also acts as a crucial bridge between the innate and adaptive immune systems by promoting B-cell differentiation and regulating T-cell immunity [2,3].

The complement system is initiated by one of three pathways, termed the classical pathway (CP), lectin pathway (LP), and alternative pathway (AP), which are defined by their mode of pattern recognition. The CP has traditionally been called the ‘antibody-dependent’ pathway of complement owing to its activation by antigen-bound antibodies of the IgG or IgM types. However, the CP is also activated by binding of the complement protein C1q to a diverse set of microbial and apoptotic cell surface structures [4]. C1q serves as the pattern recognition molecule of the CP and is itself an integral subunit of the CP initiator zymogen known as the first component of human complement, C1. C1 is a large (~790 kDa) multiprotein complex formed by the interaction of one C1q molecule, two C1r protease molecules and two C1s protease molecules. In the absence of calcium, C1 quickly dissociates, as calcium ions stabilize the formation of the heteromeric C1r_2_C1s_2_ complex as well as its interaction with C1q. Binding of C1q to pathogens or altered-self surfaces triggers the autocatalysis of C1r which in turn cleaves C1s proenzyme to form fully activated C1 [5]. In contrast, the LP is initiated via recognition of pathogen-associated molecular patterns (PAMPs) by mannan-binding lectin (MBL) or ficolins, which in turn bind and autoactivate mannan-binding lectin associated serine proteases (MASPs) [6]. Finally, the AP is continuously activated via constitutive low-level hydrolysis of complement protein C3 [7].

Independent of initiation mode, all pathways converge at the level of complement component C3, which is cleaved by multi-component enzymes called C3 convertases. The CP C3 convertase arises when activated C1 cleaves complement component C4 and C2 forming C4bC2a. The LP intersects with the CP at this step as activated MASP-2 also cleaves C4 and C2. On the other hand, the predominant AP C3 convertase (C3bBb) is formed by interaction of C3b, complement factor B, and the protease complement factor D. As their name implies, C3 convertases bind and cleave C3 resulting in the proteolytic release of the small soluble anaphylotoxin C3a, which acts to stimulate recruitment of neutrophils, monocytes and macrophages to the sites of activation. The second byproduct of C3 cleavage, C3b, covalently attaches to surfaces near the activation site where it serves as the central opsonin of the cascade eventually leading to the downstream effector functions of complement, including direct killing by formation of the terminal complement complex which has also been termed the membrane attack complex.

Host cells express several soluble and membrane associated proteins collectively referred to as regulators of complement activation, which are critical in preventing complement attack on healthy host tissue. For example, the CP is down regulated on host cells by the action of C4b-binding protein which interferes with the formation as well as accelerates the decay of the CP/LP C3 convertase while simultaneously serving as a cofactor for the complement factor I mediated degradation of C4b [8]. Complement factor H serves an analogous role in the regulation of the AP C3 convertase and in doing so acts as the dominant negative regulator of complement amplification [9]. Blood-borne pathogens, or those that traffic in interstitial fluid or lymphatics, which lack endogenous regulators of complement activity, must adopt strategies to successfully evade complement attack. One such pathogen, *Borrelia burgdorferi*, is the etiologic agent of Lyme disease and is the leading cause of vector-borne illness in the United States according to the Centers for Disease Control and Prevention (CDC). Lyme disease is often accompanied by a local erythema migrans lesion and can lead to severe clinical outcomes such as carditis, neurological dysfunction, and arthritis [10,11]. *B*. *burgdorferi* is transmitted to humans via the bite of infected hard ticks. During the ticks blood meal spirochetes enter the mammalian host and subsequently disseminate to remote tissues [10,11]. If therapeutic intervention is not sought, *B*. *burgdorferi* is able to persistently colonize a large number of tissues including joint, skin, heart, and the central nervous system [10,11]. *B*. *burgdorferi* appears to avoid complement-mediated killing from the AP by expressing a group of virulence factors known as Csp proteins (CspA and CspZ) and those from the OspE/F family [12–18]. These proteins are also referred to as complement regulator-acquiring surface proteins (CRASPs) [19,20]. These bacterial surface proteins recruit human factor H, factor H-like protein 1, and factor H-related proteins, which serve as the major endogenous negative regulators of the AP [12,13,20–23]. In addition, human factor H is also recruited to the surface of relapsing fever *Borrelia* spp. where similar AP inhibition would occur [24,25]. By hijacking these key host complement regulatory molecules, *B*. *burgdorferi*, as well as and other *B*. *burgdorferi sensu lato* isolates, subverts the deleterious effects of AP activation.

Activation of the CP has previously been shown for Lyme disease spirochetes [26,27] and studies employing mouse models deficient in factor H, factor B, or C3 have shown that the CP and/or LP play significant roles in controlling early stages of borrelial infection [28]. Indeed, the importance of spirochetal strategies to subvert CP activation are underscored by the ability of *B*. *burgdorferi* as well as the relapsing fever spirochetes *B*. *recurrentis* and *B*. *duttonii* to recruit the host CP regulators C4b-binding protein and/or C1 esterase inhibitor (C1-INH) to their surface via interactions with specific borrelial lipoproteins [29–31]. Herein we report the identification of the borrelial lipoprotein BBK32 as a potent and specific inhibitor of the CP capable of forming high-affinity interaction with C1. We go on to localize the anti-complement activity of BBK32 to the C-terminal region and demonstrate a molecular mechanism by which BBK32 noncovalently inactivates the central CP initiating serine protease C1r. To our knowledge, BBK32 represents the first example of a C1r specific inhibitor of biomolecular origin and is the first noncovalent protein inhibitor of the C1 complex to be described. Thus, this work significantly expands our knowledge of how pathogens recognize and evade human innate immunity by defining a new mechanism by which the pathogen *B*. *burgdorferi* prevents activation of the classical pathway of complement.

## Results

### The *Borrelia burgdorferi* lipoprotein BBK32 interacts with the first component of human complement, C1

**Fig 1:**
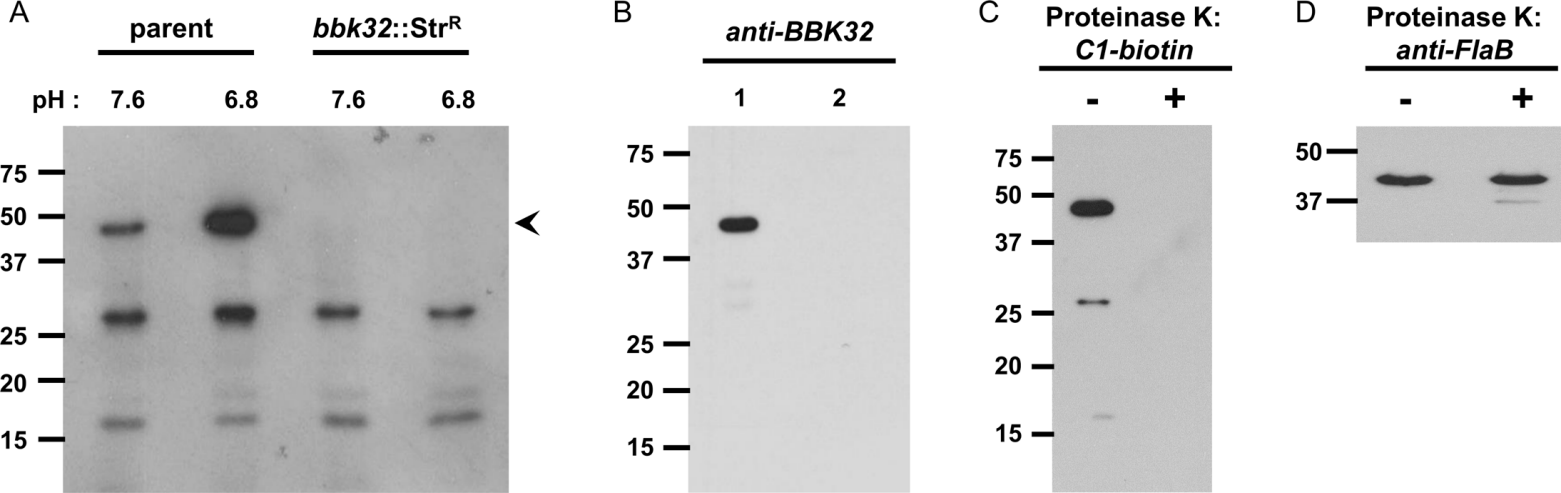
*B*. *burgdorferi* expresses surface-exposed proteins that bind complement C1. Far Western Blot analysis of *B*. *burgdorferi* cell lysates probed with complement C1. (A) Biotinylated C1 was used to probe cell lysates from *B*. *burgdorferi* B31 strain ML23 grown under conventional (pH 7.6) or induced (pH 6.8) conditions. Proteins of apparent molecular weights 17, 28, and 48 kDa were capable of binding C1, while the 48-kDa band alone was inducible. Lysates from a strain lacking an intact *bbk32* locus (*bbk32*::Str^R^) lacked the 48-kDa band. (B) Immunoblot analysis with anti-BBK32 demonstrated that the 48-kDa protein co-migrates with the C1 reactive species in the parent strain (lane 1) and is missing from the *bbk32*::Str^R^ mutant (lane 2) providing further support that it is BBK32. Both strains shown here were grown at 37°C, pH 6.8, e.g., under inducing conditions. (C) To determine if the C1 binding proteins present in borrelial lysates were surface exposed, a proteinase K assay was employed. All three bands are absent from proteinase K treated samples. (D) The subsurface endoflagellar protein FlaB are identical between mock and protease treated cells, indicating that the *B*. *burgdorferi* cells retained structural integrity.

In light of the apparent ability of *B*. *burgdorferi* to suppress the CP (discussed above), we hypothesized that novel interactions exist between *B*. *burgdorferi* surface proteins and the CP initiating enzyme complex, complement C1. To explore this hypothesis we adopted a Far Western approach designed to probe for interaction of *B*. *burgdorferi* B31 lysate proteins with C1. The initial profile showed that biotinylated C1 specifically recognizes borrelial proteins with apparent molecular masses of 17, 28, and 48 kDa (Fig 1A). We also tested the reactivity of lysates harvested from cultures grown under conditions that require the Rrp2-RpoN-RpoS regulatory system for their induction [32–35] (e.g., pH 6.8 relative to conventional growth conditions of pH 7.6) and thus mimic, in part, some aspects of the mammalian host environment [32–37] that stimulate *bbk32* expression. Given that the 48-kDa band was near the known SDS-PAGE migration position of the borrelial lipoprotein BBK32 [38], we also tested lysates originating from cells that lacked an intact *bbk32* locus (*bbk32*::Str^R^; [39]) (Fig 1A). These experiments show that the 48-kDa band is inducible under mammalian host-like conditions and is also completely absent from *bbk32*::Str^R^ lysates. The two lower molecular weight bands did not change in intensity in response to altered growth conditions and remained present in the *B*. *burgdorferi* lacking intact *bbk32*, indicating that they were not proteolytic fragments of BBK32 and instead are distinct protein species (Fig 1A). Immunoblot analysis also confirmed that the 48-kDa C1-reactive species co-migrated with BBK32 in the parent strain and was missing in the *bbk32*::Str^R^ mutant (Fig 1B).

Next, we were interested in determining if the C1-binding proteins were surface exposed in *B*. *burgdorferi*. To this end, we used a proteinase K accessibility assay that serves as a readout for the surface localization of borrelial proteins [40,41]. The results indicated that all three C1 reactive proteins were eliminated following protease treatment suggesting that they are all on the outer surface of *B*. *burgdorferi* (Fig 1C). To address the integrity of the *B*. *burgdorferi* cells, we tested whether the subsurface endoflagellar structural protein FlaB was affected by the addition of proteinase K. The levels of FlaB between mock and protease treated cells were not different (Fig 1D), indicating that the *B*. *burgdorferi* cells used in this experiment were structurally intact.

### The C-terminal globular domain of BBK32 binds C1 with high-affinity in a calcium-dependent manner

**Fig 2:**
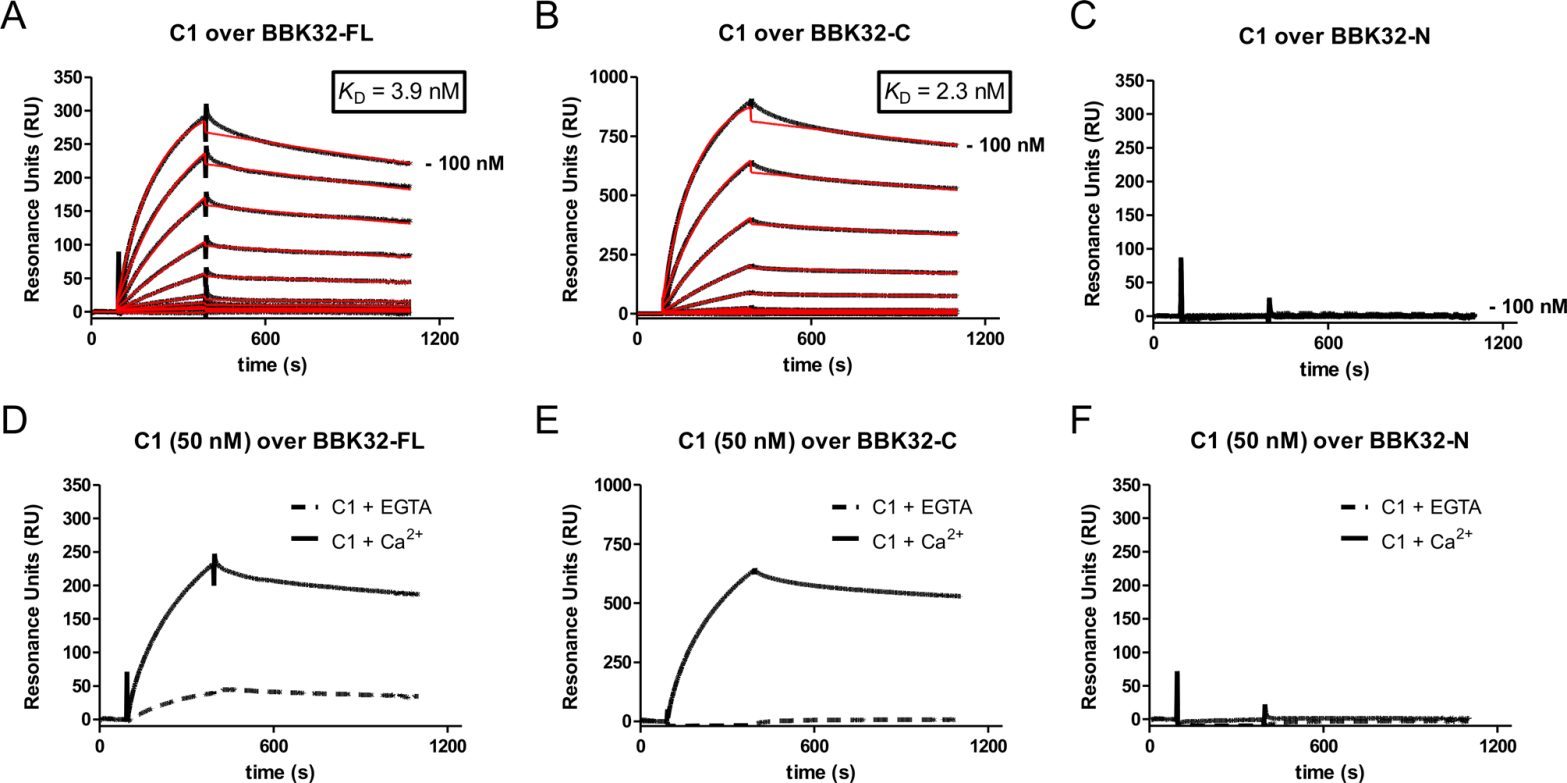
The C-terminal domain of BBK32 mediates a high-affinity and calcium-dependent interaction with complement C1. SPR was used to measure the direct binding of C1 to BBK32 proteins. A twofold dilution series of C1 (0.2–100 nM) was injected over immobilized (A) BBK32-FL, (B) BBK32-C, or (C) BBK32-N in a running buffer of HBS-T-Ca^2+^ and the SPR response was recorded (black lines). BBK32-FL and BBK32-C bind C1 with high affinity while BBK32-N exhibits no detectable response. Kinetic analysis of the resulting sensorgrams was performed and fits are represented as red traces. The derived binding constants and fitting statistics are reported in Table 1. Sensorgrams from a representative injection series are shown and all experiments were conducted in triplicate. BBK32/C1 interaction is strongly calcium dependent as evidenced by greatly diminished binding in the presence of the calcium chelator EGTA (D-F).

The identity of the 17 and 28-kDa proteins targeted by biotinylated C1 in the Far Western assay remain unclear at this time. However, in addition to gel migratory position, three lines of evidence suggested that the identity of the 48-kDa band was indeed BBK32; (i) it is induced under mammalian-like conditions (Fig 1A), (ii) it is surface exposed (Fig 1C), and (iii) it is not detected in samples containing the *bbk32*::Str^R^ allele (Fig 1A and 1B). To further investigate a potential C1/BBK32 interaction we next produced a recombinant form of BBK32 (residues 21 to 354) which lacks only the 20 residue signal peptide [38], and immobilized this “full-length” BBK32 (referred to as BBK32-FL hereafter) protein on the surface of a Biacore sensor chip. Surface plasmon resonance (SPR) was used to quantitatively measure the interaction of purified C1 with immobilized BBK32-FL in a running buffer of HBS-T-Ca^2+^. Strong and dose-dependent binding was observed (Fig 2A) and kinetic evaluation of the resulting sensorgrams indicates that BBK32-FL binds C1 with high affinity, as a dissociation constant (*K*_D_) of 3.9 nM (Table 1) was calculated.

**Table 1:** SPR: BBK32/C1 and C1r binding parameters.

Immobilized Ligand	Analyte	*K*_D_ (nM)	*k*_a_ (M^-1^s^-1^)	*k*_d_ (s^-1^)
BBK32-FL	C1	3.9 ± 0.31	(7.2 ± 0.24) x 10^4^	(2.8 ± 0.14) x 10^−4^
	C1r enzyme	15 ± 7.5	(1.9 ± 1.1) x 10^4^	(2.2 ± 0.85) x 10^−4^
BBK32-C	C1	2.3 ± 0.11	(8.4 ± 0.21) x 10^4^	(1.9 ± 0.01) x 10^−4^
	C1r enzyme	32 ± 11	(3.9 ± 2.2) x 10^3^	(1.1 ± 0.46) x 10^−4^

The dissociation constant (*K*_D_), association rate constant (*k*_a_) and dissociation rate constant (*k*_d_) were calculated by performing kinetic analysis for each interaction. All experiments were performed between three and five times and errors are reported as the mean ± SEM.

BBK32 has long been known for its ability to bind the extracellular matrix glycoprotein fibronectin (Fn) [38,42,43]. The Fn/BBK32 interaction is primarily mediated by anti-parallel *β*-strand addition of residues 126–190 originating from the intrinsically disordered N-terminal region of BBK32 with Fn type I domains along an extended region of Fn [42,44]. In addition to Fn, glycosaminoglycans (GAGs) also act as a mammalian host ligand for BBK32 and are recognized by a distinct N-terminal binding site (BBK32 residues 45–68) [45]. In contrast, the C-terminal region of BBK32 (residues 206–354 and hereafter referred to as BBK32-C) is a highly basic globular domain rich in α-helical secondary structure [46], which to date has not been ascribed a specific function. To determine if the binding site for C1 on BBK32 could be localized to the N-terminal region of BBK32 (residues 21–205, and hereafter referred to as BBK32-N) or the globular C-terminal region, we next immobilized recombinant versions of BBK32-N and BBK32-C on the surface of an SPR sensor chip. Intriguingly, no detectable response was measured when C1 was injected over the BBK32-N fragment (Fig 2C); however, the C-terminal domain of BBK32 retained a high-affinity for C1 (*K*_D_ = 2.3 nM) (Fig 2B, Table 1). Furthermore, BBK32-C has a very similar kinetic profile (*k*_a_ = 8.4 x 10^4^ M^-1^s^-1^, *k*_d_ = 1.9 x 10^−4^ s^-1^) to that measured for C1/BBK32-FL (*k*_a_ = 7.2 x 10^4^ M^-1^s^-1^, *k*_d_ = 2.8 x 10^−4^ s^-1^). The interaction of both BBK32-FL and BBK32-C with C1 was strongly dependent on calcium as binding was nearly abolished when C1 was injected in a buffer containing the calcium chelator ethylene glycol tetraacetic acid (EGTA) (Fig 2D–2F). Taken together, these results demonstrate that the C-terminal region of BBK32 forms a tight calcium-dependent interaction with human complement C1.

### The C-terminal domain of BBK32 specifically and potently blocks the classical pathway of human complement

**Fig 3:**
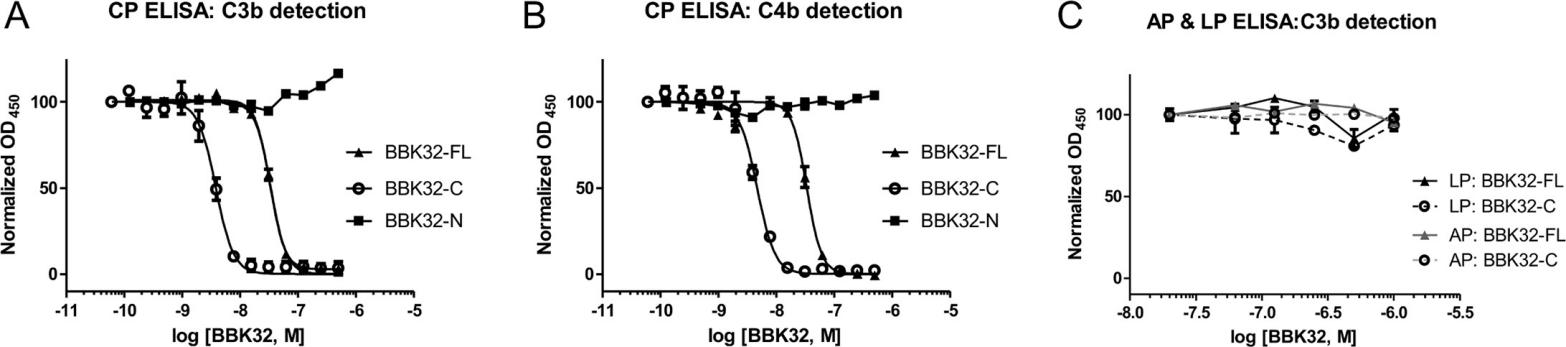
The C-terminal domain of BBK32 specifically inhibits the CP in a dose-dependent manner in an ELISA based assay of complement function. CP was selectively activated by immobilization of human IgM followed by incubation of 1% human serum in GHB^++^ buffer in the presence of varying concentrations of BBK32 proteins. Monoclonal antibodies were used to detect the deposition of the complement activation products (A) C3b or (B) C4b. While BBK32-FL and the BBK32-C potently inhibit the CP in a dose-dependent manner, BBK32-N showed no apparent inhibitory activity. In contrast, when conditions were used to select for (C) AP activation (LPS coating, 20% serum, GHB°, Mg-EGTA) or LP activation (mannan coating, 1% serum, GHB^++^) no significant inhibition was detected for up to 1 μM concentrations of any BBK32 protein derivative. Wells containing serum only or where serum was replaced with buffer were treated as 100% and 0% signal, respectively. All experiments were performed a minimum of three times, errors are reported as the mean ± SEM, and calculated IC_50_ values are reported in Table 2.

Given the apparent high-affinity interaction between BBK32 and C1 we sought to determine if BBK32 modulates complement activation. To this end, we evaluated the effect of various concentrations of BBK32-FL, BBK32-N and BBK32-C in an ELISA-based assay of complement function. When conditions specific for CP activation were used, both BBK32-FL (IC_50, C3b_ = 34 nM, IC_50, C4b_ = 34 nM) and BBK32-C (IC_50, C3b_ = 4.7 nM, IC_50, C4b_ = 5.6 nM) potently inhibited the generation of the downstream complement activation products C3b and C4b (Fig 3A and 3B, Table 2). In contrast, BBK32-N failed to inhibit CP activation at any concentration used. Importantly, when conditions were used to selectively activate the AP or LP, no significant effect could be measured at concentrations up to 1 μM of BBK32-FL or BBK32-C (Fig 3C).

**Table 2:** IC_50_ values for inhibition of CP by BBK32 proteins.

Inhibitor	Assay	IC_50_ (nM)	R^2^
BBK32-FL	ELISA: C3b detection	34 ± 2.2	0.997, 0.997, 0.996
	ELISA: C4b detection	34 ± 3.6	0.948, 0.987, 0.969
	Hemolytic	110 ± 11	0.995, 0.995
	C1r enzyme activity	670 ± 280	0.982, 0.976
BBK32-C	ELISA: C3b detection	4.7 ± 2.2	0.970, 0.961, 0.971
	ELISA: C4b detection	5.6 ± 2.0	0.927, 0.989, 0.983
	Hemolytic	60 ± 4.9	0.977, 0.987, 0.967, 0.946
	C1r enzyme activity	540 ± 91	0.983, 0.956, 0.965, 0.998
BBK32-N	ELISA: C3b detection	No inhibition	NA
	ELISA: C4b detection	No inhibition	NA
	Hemolytic	No inhibition	NA
	C1r enzyme activity	No inhibition	NA

IC_50_ values were obtained by nonlinear regression analysis using a four-parameter variable slope fit and constraining the bottom and top values to 0 and 100 respectively. The ‘goodness of fit’ parameter R^2^ is provided for each individual fit of replicate experiments. NA = not applicable.

**Fig 4:**
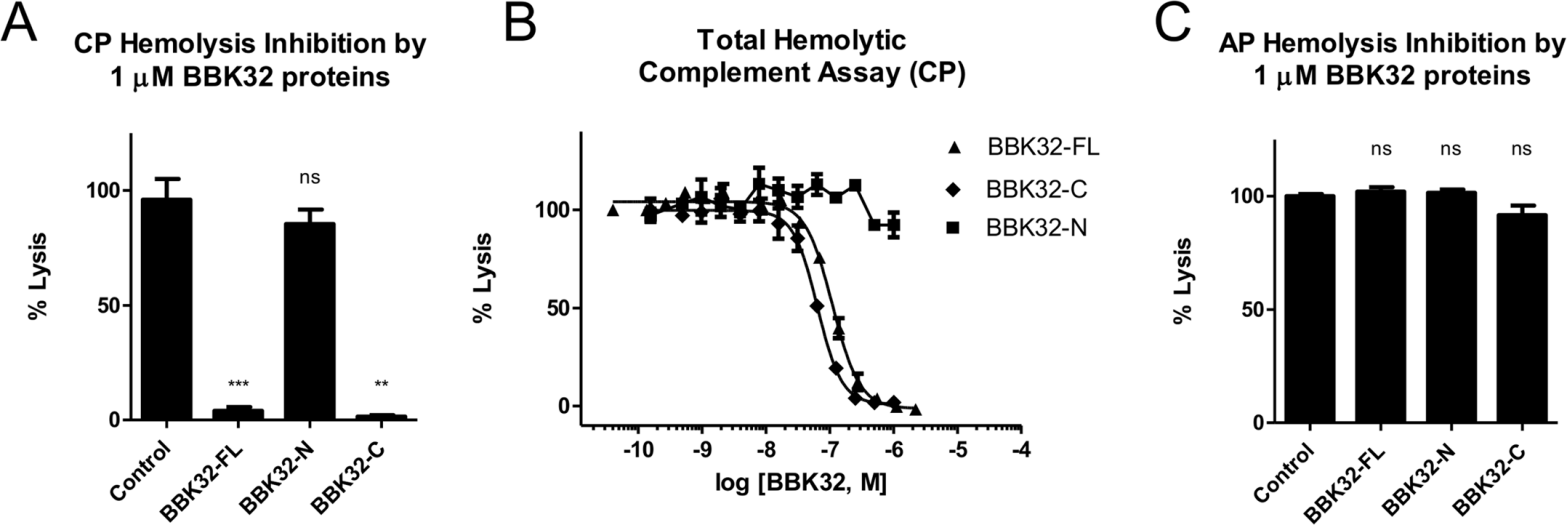
The C-terminal domain of BBK32 specifically inhibits CP-mediated hemolysis. (A) The effect of 1 μM BBK32 proteins on CP-mediated hemolysis was assessed using a standard assay of complement hemolytic function (CP50). (B) While BBK32-N exhibited no measurable effect, BBK32-FL and BBK32-C abrogated nearly all hemolytic activity in a dose-dependent manner. (C) In contrast, BBK32 proteins fail to inhibit AP-mediated hemolysis. Measures of statistical significance in (A) and (C) were determined by use of an unpaired *t* test of each experimental series versus buffer control. ** *P* ≤ 0.01, *** *P* ≤ 0.001; ns, not significant. All experiments were performed between two and four times and errors are reported as the mean ± the standard error of the mean (SEM). Calculated IC_50_ values for the dose-dependent inhibition of CP hemolysis are reported in Table 2.

To further investigate their complement inhibitory activities we next measured residual complement-mediated hemolysis in the presence of 1 μM BBK32 proteins. Strikingly, 1 μM BBK32-FL or BBK32-C conferred nearly 100% protection to sensitized sheep red blood cells in the presence of complement (i.e., normal human serum) using a standard assay of CP-mediated hemolysis (CP50) (Fig 4A). Furthermore, BBK32 inhibited the CP in a dose-dependent manner with calculated IC_50_ values of 110 nM and 60 nM for BBK32-FL and BBK32-C, respectively (Fig 4B and Table 2). Similar inhibition of the CP could not be detected for the N-terminal BBK32 fragment (Fig 4A and 4B). Consistent with the ELISA based complement assay (Fig 3C), BBK32 proteins did not have an effect on AP-mediated hemolysis of rabbit blood cells (Fig 4C). Taken together, these results demonstrate that BBK32 acts as a potent and specific inhibitor of the classical pathway of human complement and this inhibitory activity locates to the C-terminal globular region of BBK32.

### BBK32 interacts directly with the C1 subunit, C1r protease, in a calcium-dependent manner

**Fig 5:**
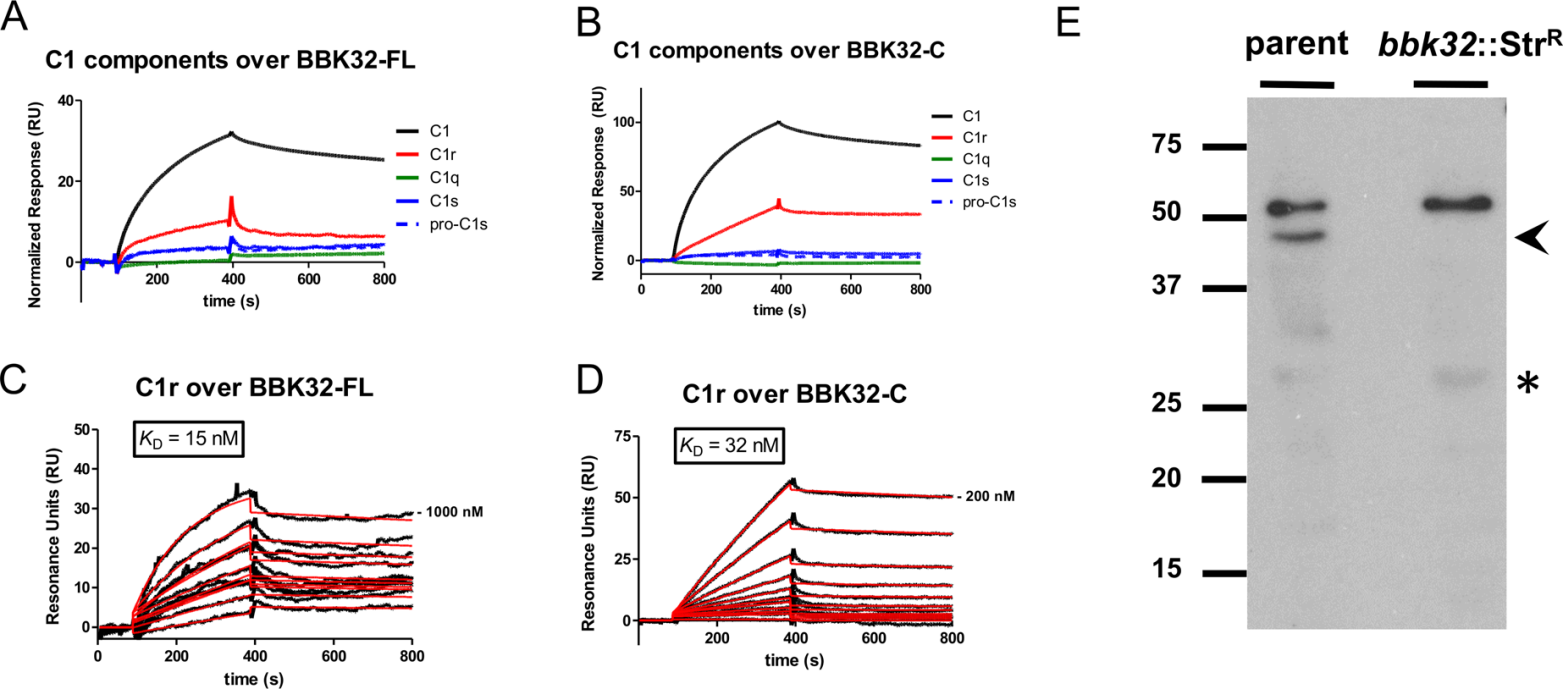
BBK32 forms a calcium-dependent, high-affinity interaction with the C1 subunit, complement C1r. SPR analysis was used to evaluate C1 component binding to BBK32-FL or BBK32-C. C1 or individual protein constituents of the C1 complex were injected at a fixed 50 nM concentration over the surface of immobilized (A) full-length BBK32-FL or (B) BBK32-C. As SPR signal is directly proportional to molecular weight, the resulting sensorgrams were normalized using the molecular weight of each analyte species. Only injection of C1 or C1r enzyme resulted in significant binding. The SPR response (black lines) was recorded for a twofold dilution injection series of C1r enzyme in a running buffer of HBS-T-Ca^2+^ over immobilized (C) BBK32-FL or (D) BBK32-C. Five independent injection series were collected and sensorgrams shown are from a representative experiment. Red traces represent fits from kinetic evaluation of the injection series. The derived binding constants and fitting statistics are reported in Table 1. (E) Far Western Blot analysis was used to assess interaction of *B*. *burgdorferi* lysates with C1r enzyme. Biotinylated C1r enzyme was used to probe cell lysates from *B*. *burgdorferi* B31 strain ML23 grown under conventional (pH 7.6) conditions. Proteins of apparent molecular weights 28, and 48, and 54 kDa were capable of binding C1r in the parent strain, while the 48-kDa band alone was absent from the strain lacking an intact *bbk32* locus (*bbk32*::Str^R^). An arrowhead denotes the band corresponding to BBK32 while an asterisk denotes a band migrating to a position observed in the C1 Far Western (Fig 1A).

The data presented above indicate that BBK32 can form a high-affinity interaction with C1 and that this interaction results in a specific inhibition of the classical pathway of human complement. The calcium-dependence of the C1/BBK32 interaction (Fig 2D–2F) raised the distinct possibility that BBK32 was capable of recognizing only the fully formed calcium-mediated C1 complex rather than binding to an individual C1 subunit with high-affinity. To determine if this was the case we used SPR and monitored the response generated by individual 50 nM injections of C1q, C1r enzyme, C1s enzyme, and C1s proenzyme over the surface of a BBK32-FL or BBK32-C SPR biosensor (Fig 5A and 5B). Surprisingly, we found that BBK32 binds specifically to C1r, but not to other C1 components (Fig 5A and 5B). In agreement with C1/BBK32 binding activity, BBK32-C binds C1r with similar affinity to that of full-length BBK32 (*K*_D, C1r/BBK32-FL_ = 15 nM vs. *K*_D, C1r/BBK32-C_ = 32 nM) (Fig 5C and 5D and Table 1). The ability of BBK32 to interact with C1r could also be observed using a Far Western approach where *B*. *burgdorferi* lysates were probed with biotinylated C1r (Fig 5E). A band corresponding to the 47-kDa BBK32 protein is detected in the parent lysate but is absent in the lysate from the *bbk32* mutant strain. Intriguingly, two additional “non-BBK32” bands are also capable of interacting with C1r using this technique. While the identities of these proteins are currently unknown, we note the presence of a 28-kDa-reactive band present in the parent strain (denoted with an asterisk) that matches the migration position of a C1 reactive band (Fig 1).

Kinetic evaluation of the SPR data suggest that both the BBK32-FL and the BBK32-C proteins recognize C1 preferentially over C1r with an increased affinity being attributed primarily to an increase in the association rate (Table 1). Nonetheless, these binding data strongly suggest that the major BBK32 binding site on the C1 complex is mediated by C1r. Interestingly, we found that the C1r/BBK32 interaction was also dependent on calcium (S1A and S1B Fig). C1r itself possesses multiple calcium binding sites and the structural and functional consequences of C1r/calcium binding are complex [47–50]. For example, calcium binding of C1r results in large-scale conformational changes of the C1r CUB2 domain from a disordered structure in the absence of calcium to a fully folded structure in its presence [49]. The dependence of the BBK32/C1r interaction on calcium suggests that BBK32 recognizes a calcium-dependent C1r conformation, which further implicates the calcium-dependent C1 complex as the physiologically relevant BBK32 ligand.

### BBK32 inhibits C1r autoactivation and prevents the enzymatic cleavage of C1s proenzyme

**Fig 6:**
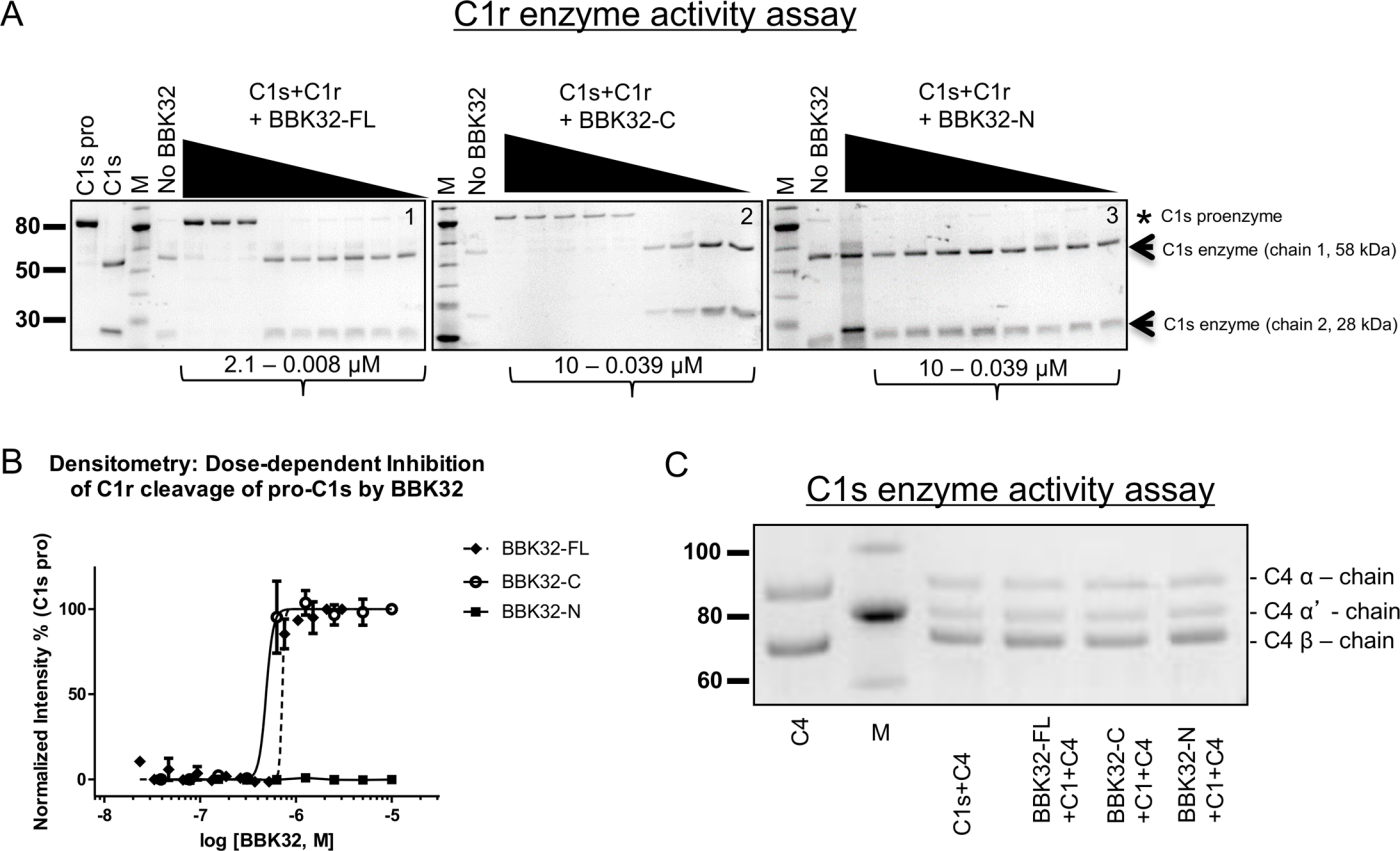
BBK32 inhibits the enzymatic cleavage of C1s proenzyme by C1r enzyme. (A) The ability of BBK32 to inhibit the *in vitro* proteolytic cleavage of C1s proenzyme by 50 nM of activated C1r enzyme was assessed by monitoring for the presence of C1s proenzyme (single 86-kDa chain; asterisk) or activated C1s enzyme (58-kDa chain 1 and 28-kDa chain 2; arrows) by SDS-PAGE. Overnight reactions were incubated at 37°C in HBS-Ca^2+^ in the presence of various concentrations of BBK32-FL (gel 1), BBK32-C (gel 2), or BBK32-N (gel 3). In the absence of BBK32, C1r enzyme converts 100% of C1s proenzyme to C1s enzyme under the conditions used (see “No BBK32” lanes on gels 1–3). BBK32-FL and BBK32-C inhibited C1r activity in a dose-dependent manner, whereas, BBK32-N failed to inhibit C1r up to a final concentration of 10 μM. All C1r activity assays were conducted a minimum of three times and representative gel images are shown. (B) Densitometry was performed and the normalized peak intensity of the band corresponding to C1s proenzyme was plotted against the concentration of BBK32 present in each reaction. IC_50_ values were calculated and are reported in Table 2 along with statistics for individual fits. (C) The ability of 1 μM BBK32 proteins to inhibit the cleavage of complement C4 by previously activated and purified C1s enzyme was evaluated *in vitro* by monitoring the conversion of the C4 α-chain to the C4 α’-chain by SDS-PAGE. C1s enzyme activity could not be detected under the conditions used for any BBK32 proteins, indicating that the BBK32 inhibitory activity is specific for C1r. C1s activity assays were performed in duplicate and a gel image from a representative experiment is shown.

The ability of BBK32 to bind both C1 and C1r and to specifically inhibit the classical pathway of complement suggested that BBK32 interfered with the enzymatic activity of the C1r protease. To determine if this was the case we first measured the effect of various concentrations of BBK32-FL, BBK32-C, and BBK32-N on the *in vitro* conversion of C1s proenzyme by previously activated C1r enzyme (Fig 6A). Indeed, both BBK32-FL and BBK32-C inhibited C1r in dose-dependent fashion, while BBK32-N failed to inhibit up to a 10 μM final concentration. Quantification of these data by densitometry was performed by evaluating the peak intensity of the C1s proenzyme band (Fig 6B) and calculated IC_50_ values are reported in Table 2. BBK32 proteins do not affect the activity of previously activated C1s (C1s enzyme) as C4 is cleaved by C1s in the presence of 1 μM BBK32 proteins (Fig 6C). These results indicate that BBK32 specifically inhibits C1r enzyme by preventing the processing of its natural substrate, the C1s proenzyme.

**Fig 7:**
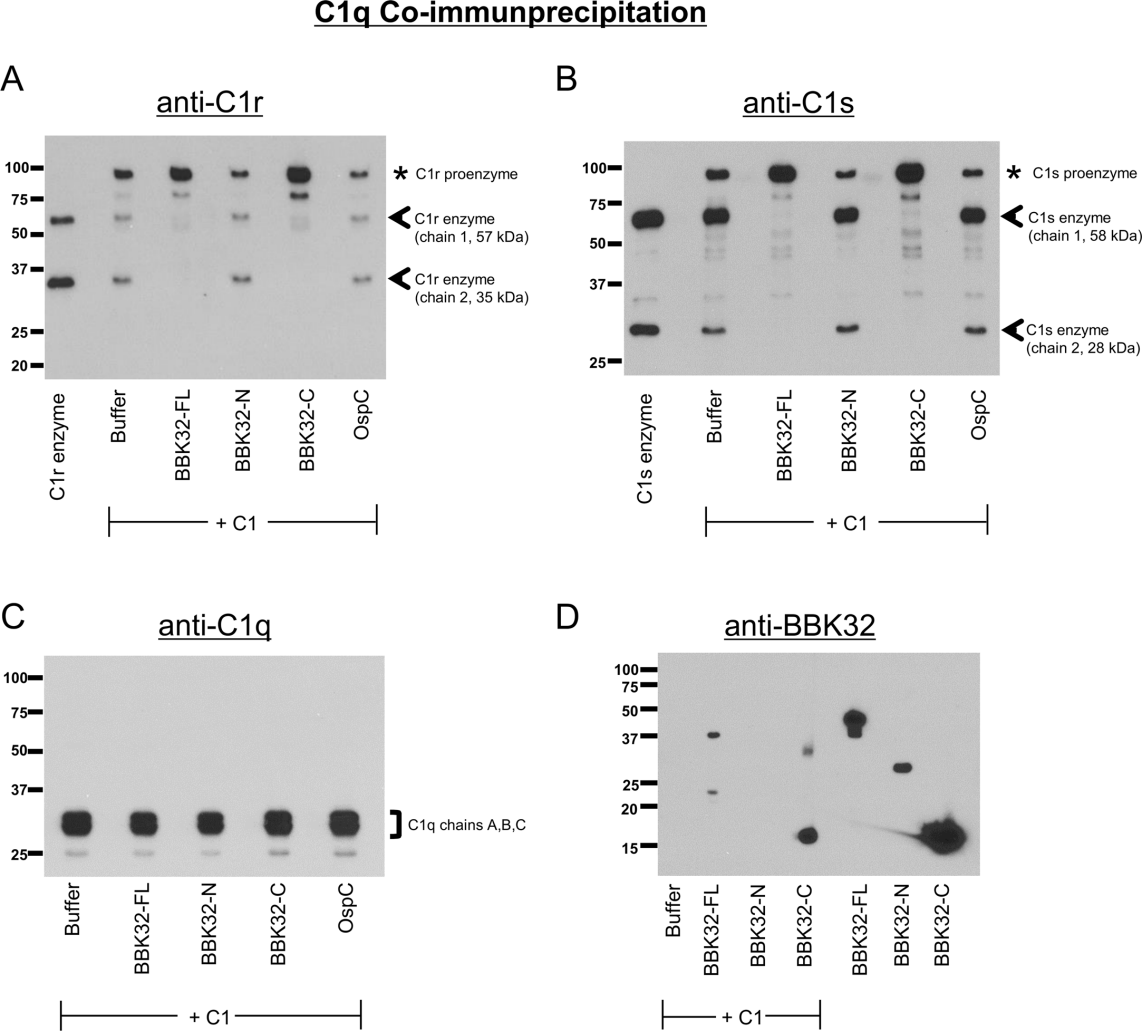
BBK32 inhibits the autocatalysis of C1r within the C1 complex. C1 (40 nM) was incubated at 37°C for 2 hours in the presence or absence of 5 μM BBK32 or OspC proteins. Reactions were co-immunoprecipitated using a C1q monoclonal antibody previously adsorbed to protein G beads, and bound fractions were subjected to Western immunoblot analysis. (A) Reactions were probed with C1r polyclonal antibody. Previously purified activated C1r enzyme is loaded as a reference. Activated C1r is detected in buffer only reactions as judged by the presence of C1r enzyme chain 1 (57 kDa) and chain 2 (35 kDa), denoted by arrowheads. Reactions incubated with BBK32-FL or BBK32-C lack processed C1r and contained only the 92 kDa C1r proenzyme form (denoted by an asterisk). BBK32-N and a negative control protein (OspC) reactions contain processed C1r at levels indistinguishable from the buffer only reaction. (B) Reactions were probed with a C1s polyclonal antibody and previously purified activated C1s is loaded for reference. As was observed for C1r, reactions incubated with BBK32-FL or BBK32-C lack activated C1s and contain only C1s proenzyme (asterisk). Buffer only, BBK32-N, and OspC reactions contain equal amounts of activated C1s as judged by the presence of C1s enzyme chain 1 (58 kDa) and chain 2 (28 kDa) (denoted with arrowheads). (C) Detection with C1q polyclonal antibody indicates equivalent amounts of C1q are pulled down in all reactions. (D) Detection with BBK32 polyclonal antibody demonstrates that BBK32-FL or BBK32-C but not BBK32-N are pulled down with the C1 complex. The last three rightmost lanes contain 100 ng of each form of BBK32 protein as a reference.

When C1 is incubated at 37°C, it becomes activated by the autocatalysis of C1r proenzyme and subsequent C1r enzyme cleavage of the C1s proenzyme [51]. To determine if BBK32 affects C1r and C1s zymogens within the C1 complex, we incubated BBK32 proteins (5 μM) with C1 (40 nM) for two hours at 37°C and co-immunoprecipitated (co-IP) C1 using a C1q monoclonal antibody. Bound fractions were subjected to SDS-PAGE, followed by Western immunoblot analysis, and the presence of C1r, C1s, C1q, or BBK32 was then assessed. When reactions were probed with C1r antibody (Fig 7A), a processed form of C1r was observed for the buffer only control as indicated by the presence of C1r chain 1 (57 kDa) and C1r chain 2 (35 kDa). Interestingly, reactions containing BBK32-FL or BBK32-C contain only the proenzyme form of C1r (92 kDa). In contrast, reactions incubated with BBK32-N or an independent negative control (recombinant borrelial lipoprotein OspC) were indistinguishable from the buffer only reaction. These results demonstrate that BBK32 inhibits the autocatalysis of the C1r proenzyme. In agreement with the presence of inactivated C1r within the C1 complex, we found essentially only C1s proenzyme (86 kDa) when we probed the reactions containing BBK32-FL or BBK32-C with the C1s polyclonal antibody (Fig 7B)_._ As would be expected by the presence of activated C1r, we found cleaved C1s in the buffer only, BBK32-N, and OspC reactions. C1q is detected at identical levels in all reactions, confirming the validity of the co-IP approach (Fig 7C), and importantly, BBK32-FL or BBK32-C but not BBK32-N were pulled down with the C1 complex (Fig 7D). Taken together, these results show that BBK32 bound C1 is trapped in an inactive form whereby the autocatalytic activation of C1r is inhibited and subsequent C1r cleavage of C1s proenzyme is blocked and that the BBK32 inhibitory effect is specific for the C-terminal half of the protein.

### *B*. *burgdorferi* binds to C1 and C1r in a BBK32-specific manner

**Fig 8:**
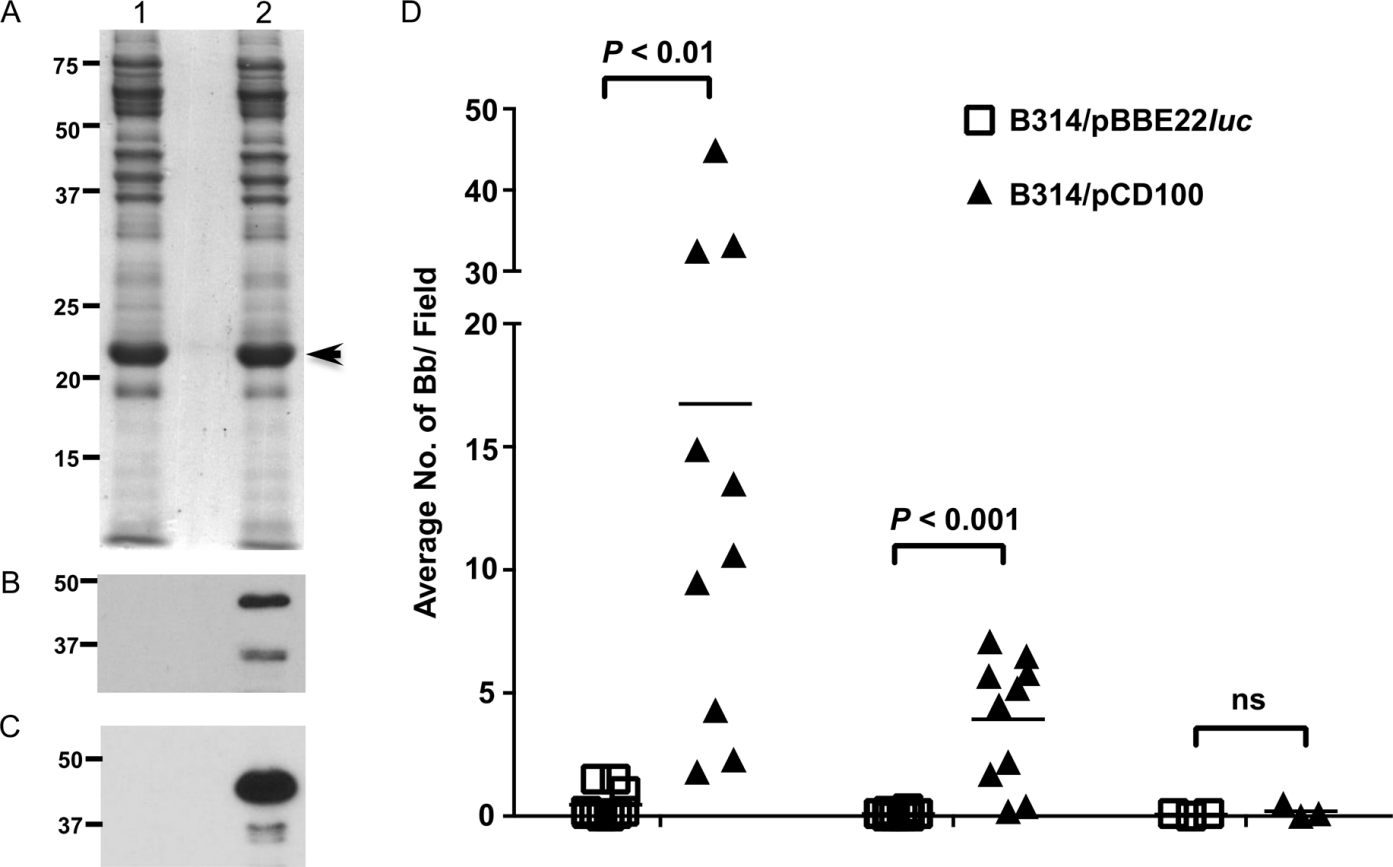
Surface exposed BBK32 promotes binding of *B*. *burgdorferi* to human C1 and C1r. Strain B314 total cell protein lysates from cells containing the vector pBBE22*luc* (lane 1) and natively expressed *bbk32* (plasmid construct pCD100; lane 2) were stained with Coomassie blue (A), immunoblotted against a monoclonal antibody specific for BBK32 (B), and subjected to Far Western overlay analysis with biotinylated C1 (C). B314/pBBE22*luc* and the *bbk32* expressing strain B314/pCD100 were incubated with immobilized C1, C1r, and BSA to assess BBK32-dependent binding to these target proteins (D). Each data point represents the average number of spirochetes present on 10 independently read fields as scored by dark field microscopy. The horizontal bar represents the mean value. Statistical significance in (D) was determined by use of a multiple *t* test followed by the Sidak-Bonferroni method to correct for multiple comparisons. All experiments were performed independently between three and five times. Significant differences are reflected in the *P* values shown. ns; not significant.

We were interested in determining if similar activities were also observed with native BBK32 produced in *B*. *burgdorferi*. To test whether surface exposed BBK32 could mediate binding to components of the classical complement pathway, we incubated infectious *B*. *burgdorferi* and a *bbk32*::Str^R^ derivative (strain JS315) with immobilized C1 (grown under conditions that induce *bbk32*). Interestingly, both the parent and the *bbk32* isogenic mutant readily bound C1 and no significant difference in binding could be detected (S2 Fig). One plausible explanation for this result is a likely layer of functional redundancy provided by the presence of additional *B*. *burgdorferi* proteins capable of C1 recognition as evidenced in the overlay analysis (Fig 1A). To provide a less complex outer surface environment for *B*. *burgdorferi*, we used strain B314, which is missing linear plasmids (lp) and thus does not synthesize many lp-encoded borrelial proteins associated with both mammalian and tick infectivity, specifically OspAB, DbpBA, and BBK32 [52,53]. A shuttle vector containing *bbk32* with its native promoter (pCD100) was transformed into strain B314, along with a vector-only control (pBBE22*luc*). An equivalent amount of protein was loaded from each strain (Fig 8A) and subsequent immunoblot and biotin-labeled C1 overlay analysis showed that, as expected, B314/pCD100 made detectable levels of BBK32 capable of binding C1 whereas B314/pBBE22*luc* did not (Fig 8B and 8C). To determine whether natively produced BBK32 mirrored the C1/C1r-binding profile of purified recombinant BBK32, we exposed these strains to immobilized C1 and C1r. Unlike the wild-type strain, the B314 control strain (B314/pBBE22luc) exhibited very little binding to C1 or C1r (Fig 8D). In contrast, the presence of BBK32 in B314/pCD100 transformed organisms promoted a significant enhancement of spirochete binding to both of these targets (Fig 8D).

### BBK32 promotes resistance to a serum sensitive *B*. *burgdorferi* strain

**Fig 9:**
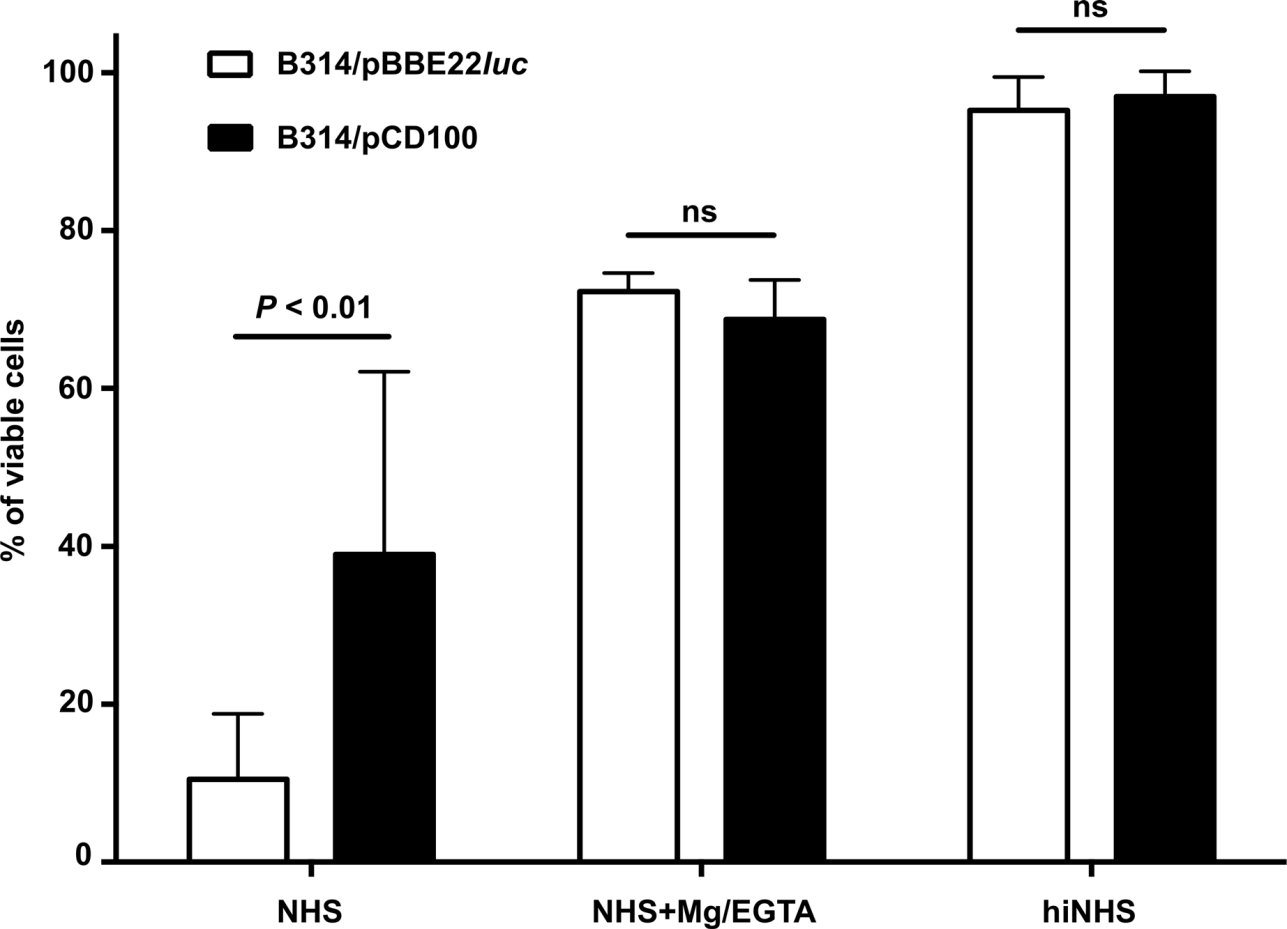
Production of BBK32 provides resistance to the serum sensitive strain B314. A *B*. *burgdorferi* strain natively expressing BBK32 (B314/pCD100) is significantly more resistant to serum-based inactivation relative to the isogenic parent strain B314/pBBE22*luc*. Conditions that selectively block the CP/LP but leave the AP intact (NHS+Mg/EGTA) enhanced survival of these cells in a BBK32-independent manner. As expected, heat inactivated serum (hiNHS) ablated all complement-dependent killing for both strains. Statistical significance was determined by use of a multiple *t* test followed by the Sidak-Bonferroni method to correct for multiple comparisons and is depicted as the mean value ± the standard deviation. All experiments were performed independently between three and five times. Significant differences are reflected in the *P* values shown. ns; not significant.

We were next interested if the production of BBK32 could promote resistance to serum. For this purpose, we again utilized *B*. *burgdorferi* strain B314, which in addition to its avirulent phenotype, is rendered serum sensitive, presumably due to its aforementioned loss of lp content [53]. We hypothesized that the selective addition of BBK32 would make strain B314 resistant to complement dependent killing and, to assess this, tested B314/pBBE22*luc* and B314/pCD100 for their relative resistance to serum. Consistent with an anti-complement activity, the presence of natively expressed BBK32 in B314/pCD100 provided significant protection against serum based immobilization relative to strain B314/pBBE22*luc* that lacked BBK32 (Fig 9). As expected, heat inactivation resulted in limited killing with either strain tested (Fig 9). When conditions were used that selectively block the CP/LP but keep the AP intact (NHS + Mg-EGTA) [27] both strains exhibited equivalent resistance/sensitivity independent of BBK32 levels (Fig 9). These data imply that a majority of the inactivation observed for serum-sensitive B314/pBBE22*luc* is due to the adverse effects of the CP/LP. Furthermore, these data strongly suggest that the BBK32-dependent protection observed with untreated serum is due to the ability of BBK32 to neutralize either the CP or LP. Coupled with the C1/C1r-specific binding profile (Figs 1, 2 and 5), CP-specific inhibition (Figs 3 and 4), and C1r inhibitory activity (Figs 6 and 7) of recombinant BBK32 proteins along with the CP-specific function of C1r [54], the serum resistance mediated by BBK32 in this assay is best explained by BBK32-dependent inactivation of the CP.

## Discussion

In order to survive the destructive forces of the human complement cascade, successful microbial pathogens have evolved a number of sophisticated evasion strategies. Although specific modes of complement recognition and inactivation are widely varied, these inhibitory mechanisms can be conceptually grouped into three forms: direct recruitment or mimicry of host regulators of complement activity such as complement factor H, enzymatic degradation of complement components by direct or indirect means, or inhibition through direct interaction with complement proteins [55]. In addition to employing each of these evasion strategies, *B*. *burgdorferi*, takes advantage of its complex mammalian-tick lifestyle by exploiting anti-complement molecules produced by the tick salivary glands during tick feeding [20]. Recruitment of factor H related molecules by the borrelial CspA, CspZ, and the OspE/F proteins has been studied extensively [12–23,56] and C4b-binding protein has been reported to interact with *B*. *burgdorferi*, *B*. *afzelii*, *and B*. *garinii* [30], while the relapsing fever spirochetes have been shown to bind factor H [24,25] C4b-binding protein and C1-INH [29,31]. In addition to their direct recruitment, an example of host complement regulator mimicry has also previously been reported for *B*. *burgdorferi* [57]. To our knowledge, the only previously reported example of a borrelial factor possessing direct and novel complement inhibition activity is the recently described terminal complement complex inhibition function of CspA [20,23]. CspA along with its ability to bind factor H/factor H like-1 and plasminogen also acts a direct inhibitor of the terminal complement complex by binding complement components C7 and C9 and interfering with C5b-9 complex assembly [23].

All three pathways of complement can be activated by *B*. *burgdorferi* and all result in direct complement-mediated killing of the spirochete [20,23]. In addition to becoming activated by immune complexes, the CP has also been shown to kill *B*. *burgdorferi* in the absence of specific antibodies [27]. The requirement for protection from complement attack for *B*. *burgdorferi* is evidenced by the production of a number of virulence factors (now to include BBK32) that specifically target and inactivate complement [20,23]. In this study we determined that the C-terminal globular region of the borrelial lipoprotein BBK32 exhibits a potent CP-specific inhibitory activity that confers serum resistance to a normally serum-sensitive *B*. *burgdorferi* strain. While the focus of this study was on elucidating the anti-complement activity and mechanism of BBK32, our probe of lysates with C1 and C1r suggest a robust interaction between *B*. *burgdorferi* and the CP may exist. Despite the linkage of *B*. *burgdorferi* to complement resistance, the small number of experimental infectivity studies employing mice deficient in key components of complement has shown a surprisingly limited role for complement in controlling *B*. *burgdorferi* burden [28,58,59]. Thus, it is of interest to consider potential roles for the inhibition of the CP beyond protection from complement-mediated attack. For example, upon colonizing lymph tissue *B*. *burgdorferi* disrupts the normal formation of germinal centers (GC) [60,61]. Lack of normal GC development ultimately results in reduced antibody titers against *B*. *burgdorferi* in experimental infection [60]. Local complement C4 deposition on follicular dendritic cells (FDC) is significantly reduced in *B*. *burgdorferi* infected lymph nodes and this is speculated to be responsible for the premature collapse of GC responses due to diminished antigen presentation by FDCs [60]. In this regard, it is of interest to determine if BBK32 mediates this lymphoid specific effect, resulting in the observed reduction in the humoral immune response to borrelial antigens. It would be of additional interest to understand the potential role of BBK32 anti-complement activity in the context of spirochetal persistence in a natural reservoir animal such as *Peromyscus leucopus* [62]. Studies to address some of these possibilities are currently underway.

**Fig 10:**
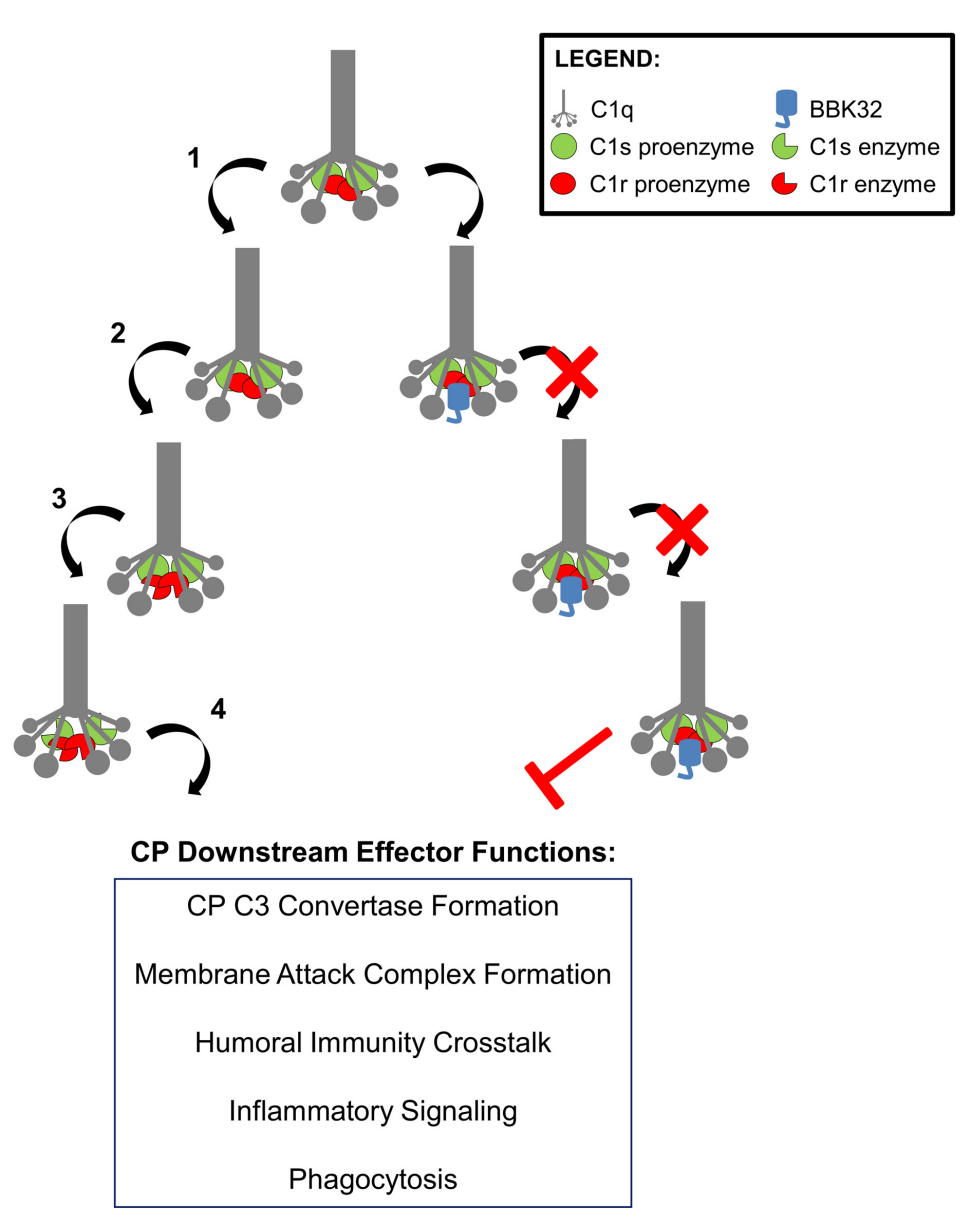
Model of C1 inactivation and CP inhibition by BBK32. C1 circulates as an inactive zymogen composed of one C1q molecule, two C1r proenzyme molecules, and two C1s proenzyme molecules. The pathway on the left depicts the normal uninhibited activation of C1 that proceeds by binding of the pattern recognition molecule C1q to a bacterial surface (1). C1q binding triggers the autocatalysis of the C1r proenzymes (2) that subsequently cleave and activate C1s (3). C1s then cleaves downstream CP components (4), which ultimately lead to many downstream CP effector functions. The pathway on the right side depicts the situation in the presence of the BBK32 lipoprotein. Following C1q binding to the borrelial surface the C-terminal domain of BBK32 recognizes C1 by binding directly to C1r. BBK32 blocks the autocatalytic and C1s proenzyme proteolysis functions of C1r (denoted in both instances by a red X). Thus, BBK32 effectively renders C1 in the zymogen form leading to abrogation of CP activation (denoted by a red bar).

In the current study we show that BBK32 acts directly by binding to and inhibiting C1 via a novel mechanism involving the noncovalent inhibition of C1r enzymatic activity. A model for the inhibition of C1 by BBK32 is depicted in Fig 10. When C1 engages a surface via C1q binding, this information is transmitted by coordinated conformational changes within the C1 complex ultimately triggering autocatalysis of C1r and subsequent activation of C1s [63]. Under physiological conditions this activation is extremely rapid [64] and controlled on the surface of host cells by the only known endogenous inhibitor of C1, C1-INH. C1-INH covalently modifies the C1r and C1s active sites and promotes their release from ligand-bound C1q [65]. C1-INH is a member of the serpin family of protease inhibitors and along with inhibiting both C1r and C1s it also inactivates a number of blood proteases involved in the complement, contact, fibrinolytic, and coagulation systems [65]. The broad protease-binding specificity and covalent inhibitory mechanism of C1-INH stands in stark contrast to that of BBK32, which specifically binds and inhibits C1r and had no detectable effect on the homologous C1s protease. It has been shown that serum deficient in C1r is unable to undergo CP activation while retaining a fully functional LP [54]. In our studies BBK32 did not block the LP or AP suggesting it is unable to inhibit the primary serine proteases of these pathways (i.e., MASPs and factor D, respectively). Instead, BBK32 specifically inactivates the CP by preventing the autocatalysis of C1r proenzyme and subsequent cleavage of C1s proenzyme ultimately rendering C1 entrapped in a zymogen form.

Since its discovery as a fibronectin and glycosoaminoglycan-binding protein expressed on the surface of *B*. *burgdorferi* [38,43], BBK32 has been the subject of intense study [39,44–46,66–68], including a recent report indicating a role in bloodstream survival [69]. In this regard, BBK32 has been shown to be critical for borrelial pathogenesis [39,68], capable of exploiting host fibronectin function [44,46,70–73], shown to be involved in borrelial vascular adhesion mechanisms [69,72,73], and in promoting joint colonization [45]. Surprisingly then, infectivity of a *B*. *burgdorferi bbk32* mutant shows a limited infectivity phenotype when experimental infection is done at a high inoculum dose (i.e., 10^5^) [39,68,74]. However, this effect is restricted to high doses, as lower inoculum doses (e.g., 10^3^) exhibit a significant reduction in colonization [39] that is most apparent using *in vivo* imaging for detection [68]. Furthermore, *bbk32* mutant strains exhibit a delay in the ability to disseminate [68]. Nonetheless, one potential explanation for the relatively mild phenotype of the *bbk32* mutant is a layer of functional redundancy present in the spirochete. For example, several borrelial proteins besides BBK32 are now known to bind directly to fibronectin [75–77], and although much remains to be known about their specific functional activities, it is possible that these proteins could overlap with BBK32’s fibronectin related functions. It seems a similar situation may exist for the BBK32 anti-complement activities described here. The presence of multiple bands in the Far Western lysate probes (Figs 1 and 5E) suggest that *B*. *burgdorferi* may express one or more “non-BBK32” proteins capable of interacting with both C1 and C1r. The existence of additional borrelial proteins able to compensate a loss of BBK32 may further explain the muted *bbk32* mutant phenotype. Determining the identity of these proteins along with evaluating their potential to interfere with the CP remains an important next step.

Orthologs of BBK32 are found not only in Lyme disease spirochetes, but also in relapsing fever spirochetes where they have been divided into three groups based on phylogenetic relationships [78]. Interestingly, the Lyme disease spirochete *B*. *valaisiana* strain ZWU3 Ny3 was shown to possess a novel mechanism of complement inhibition independent of host complement regulator recruitment/mimicry [79]. Although a molecular mechanism has not been elucidated, the authors hypothesize that the inhibitory mechanism present in this strain of *B*. *valaisiana* relies on direct interaction with complement components [79]. We note that a BLASTP search of BBK32 from *B*. *burgdorferi* B31 reveals a gene located on linear plasmid 28 of *B*. *valaisiana* strain VS116 that encodes a 113 residue hypothetical protein that contains 60% identity and 72% similarity with the C-terminus of BBK32 (residues 181 to 303). While it is not known if this is a functional gene product possessing BBK32 anti-complement activity, it is intriguing that the hypothetical protein lacks the GAG and Fn-binding sequences that are the hallmark of BBK32. Nonetheless, future studies are required to understand if the complement inhibitory activity of the BBK32 C-terminal domain is conserved across borrelial species.

In the same light, it remains to be seen if BBK32 anti-complement mechanisms are found in other human pathogens that are known to have evolved similar molecular mechanisms of host interaction. For instance, BBK32 itself shares nearly identical modes of fibronectin interaction with a group of fibronectin-binding proteins from staphylococcal and streptococcal bacterial species [44,80–83]. While these Gram-positive encoded proteins lack sequence conservation with C-terminal BBK32 sequences, it may be important to assess their potential role in complement interaction. The possibility is raised that an underlying benefit to the microorganism exists in producing proteins capable of simultaneously interacting with host extracellular matrix molecules like fibronectin and components of the complement system. Such an example of a synergistic mode of interaction exists for the extracellular fibrinogen-binding (Efb) protein expressed by *Staphylococcus aureus*. A disordered N-terminal region of Efb binds directly to human fibrinogen, while a highly basic globular domain originating from the C-terminal region of the protein binds with high affinity to complement component C3 [84–86]. Although each molecular interaction individually contributes to virulence, a ternary fibrinogen-Efb-C3b complex can form, encapsulating the bacteria in a ‘fibrinogen-shield’, which results in direct inhibition of phagocytosis [87]. While the similarity of the BBK32 molecular architecture to that of staphylococcal Efb is striking, it is currently unknown how BBK32/fibronectin binding affects BBK32 anti-complement activities or if an analogous functional synergism exists. The work presented here provides the conceptual framework to explore this and related questions on complex host-pathogen interactions involving extracellular matrix and complement proteins.

## Methods

### Bacterial strains and plasmids

*B*. *burgdorferi* B31 strains ML23 [88], JS315 (ML23 *bbk32*::Str^R^;[39]), and B314 were grown in BSK-II media supplemented with 6% normal rabbit serum (Pel-Freez Biologicals, Rogers, AR) under microaerobic conditions at 37°C, 5% CO_2_ atmosphere, at either a pH of 6.8 or 7.6. The serum sensitive strain B314 (kindly provided by Tom Schwan) is a non-infectious variant of strain B31 that lacks most linear plasmids [43,45,52,53]. All *B*. *burgdorferi* cells were enumerated by dark field microscopy.

Native *bbk32* was cloned into the shuttle vector pBBE22*luc* [68] using the following approach. The oligonucleotide primers BBK32Comp-BamHI-F (5’-ACGC**GGATCC**GTACTTTGTTCACCCTCTTGATAGC-3’; BamHI site is in **bold**) and BBK32Comp-SalI-R (5’-ACGCGTCGACATATTATGTAGCCTGTTTTAAATT-3’; SalI site is underlined) were used to PCR amplify *bbk32* from strain B31 genomic DNA. The PCR-amplified product contains 213 bp of upstream sequence and 37 bp downstream from the translational start site and stop codon of the 1,062 bp *bbk32* gene, respectively. The resulting 1.3 kb fragment was cloned into plasmid pCR-Blunt-II-TOPO and transformed in Mach1-T1R *Escherichia coli* cells (F^–^ ϕ80*lac*ZΔM15 Δ*lac*X74 *hsd*R(rK–, mK+) Δ*rec*A1398 *end*A1 *ton*A; ThermoFisher). The resulting construct was digested with BamHI and SalI and the 1.3 kb fragment subsequently cloned into BamHI and SalI cut pBBE22*luc*. The resulting construct, which contained *bbk32* expressed from its native promoter, was designated pCD100.

Transformation of strain B314 with pCD100 and pBBE22*luc* was done as previously described [89]. Transformants were selected for resistance to kanamycin and screened by PCR to confirm the presence of pCD100 using primers BBK32Comp-BamHI-F and BBK32Comp-SalI-R.

### Proteinase K accessibility assay

*B*. *burgdorferi* strain ML23 was grown under inducing conditions (37°C, 5% CO_2_, pH 6.8) and harvested by centrifugation at 5,800 x *g*, and washed twice with PBS. The cell pellet was resuspended in 0.5 ml of either PBS alone, or PBS with proteinase K (to a final concentration of 200 μg ml^-1^). All samples were incubated at 20°C for 40 min. Reactions were terminated by the addition of phenylmethylsulfonyl fluoride (PMSF) to a final concentration of 1 mM. Cells were again pelleted by centrifugation (9,000 x *g* for 10 min at 4°C), washed twice with PBS containing 1 mM PMSF, and resuspended in Laemmli sample buffer [90]. Samples corresponding to 5 x 10^7^ whole cell equivalents were run on SDS-PAGE gel, transferred to PVDF membranes and probed with biotinylated C1 in a Far Western analysis or immunoblotted with a monoclonal antibody to borrelial FlaB (Affinity BioReagents, Inc.), as described below.

### Proteins

DNA fragments encoding residues 21 to 205 or 206 to 354 of BBK32 (*B*. *burgdorferi* B31 strain) were PCR-amplified from pQE30-BBK32-FL plasmid DNA [46] using oligonucleotide primers that appended BamHI and NotI sites at the 5’ and 3’ ends, respectively. Restriction digested DNA fragments were then sub-cloned into the pT7HMT vector [91]. Sequence confirmed plasmids were transformed into *E*. *coli* strain BL21(DE3) for protein production.

Expression and purification of recombinant BBK32-FL was performed as previously described [46]. Recombinant BBK32-N and BBK32-C were overexpressed by inoculating 1 liter of Terrific Broth with 10 ml of an overnight BL21(DE3) at 37°C, induced with 1 mM isopropyl-D-thiogalactopyranoside upon reaching an optical density at 600 nm of 0.6–0.8, shifted to 18°C shaking, and allowed to express overnight. Overnight cultures were harvested by centrifugation at 5,000 x *g* for 10 min and resuspended in Ni-NTA-binding buffer (20 mM Tris (pH 8.0), 500 mM NaCl, 10 mM Imidazole (pH 8.0)) and lysed by microfluidization. A clarified cell extract was obtained by centrifugation at 25,000 x *g* for 35 min and the supernatant was applied to a nickel agarose (Gold Bio) column previously equilibrated in Ni-NTA buffer, washed with 5 column volumes (CV) of Ni-NTA-binding buffer, and eluted with 2 CV’s of Ni-NTA elution buffer (20 mM Tris (pH 8.0), 500 mM NaCl, 500 mM imidazole (pH 8.0)). The HIS-myc affinity tag was removed by enzymatic digestion with tobacco etch virus (TEV) protease in the presence of 5 mM β-mercaptoethanol at room temperature for 2 h. The digestion reaction was allowed to continue overnight at 4°C while being dialyzed against Ni-NTA-binding buffer. The TEV digested sample was then incubated with a nickel agarose column and the flow through fraction was collected and subjected to gel filtration chromatography using a HiLoad Superdex 200 PG column (GE Healthcare) equilibrated in 20 mM Tris (8.0), 200 mM NaCl. Peaks were analyzed by SDS-PAGE and fractions corresponding to BBK32 proteins were pooled and concentrated using Amicon centrifugal filters (EMD Millipore), aliquoted, and stored at -80°C until use.

Purified C1, C1r enzyme, C1q, C1s enzyme, C1s proenzyme, and C4 were obtained from Complement Technology (Tyler, TX). Human C1 or C1r proteins were biotinylated using EZ-link Sulfo-NHS-LC-Biotin (Thermo Fisher Scientific) at the molar ratio suggested by the manufacturer. The labeling reaction was quenched by the addition of Tris HCl pH 7.6 to a final concentration of 20 mM. The sample was diluted in protein-binding buffer (100 mM NaCl, 20 mM Tris pH 7.6, 1 mM EDTA, 10% glycerol, 0.2% Tween-20, 2% non-fat milk) [92] and used below in the Far-Western assay.

### Far Western immunoblotting and conventional immunoblotting

For Far Western analyses, *B*. *burgdorferi* protein lysates were resolved by SDS–PAGE [90] and gels were transferred to PVDF membranes as described [93]. The membrane was blocked in 10% non-fat milk, washed with PBS, 0.2% Tween-20, and then 20 μg biotinylated C1 or C1r (both from CompTech), at 1 μg/ml in protein binding buffer [92], was incubated with the membrane overnight at 4°C. The membrane was washed extensively in PBS, 0.2% Tween-20, and then incubated with Vectastain solution (Vectastain Elite ABC Kit, Vector Laboratories), as instructed by the manufacturer, to enhance the signal. The membrane was then washed and developed using the Western Lightning Chemiluminescent Reagent plus system (Perkin Elmer, Waltham, MA, USA).

Conventional immunoblotting was done as previously described [94]. Production of BBK32 in B314/pCD100 was evaluated using either a monoclonal antibody to BBK32 (a generous gift from Seppo Meri, University of Helsinki) diluted to 1:4000 or, in the case of the co-immunoprecipitations (see below), a polyclonal antibody against BBK32 diluted 1:1500. Immunoblots to detect the endoflagellar antigen FlaB were done using a monoclonal to *B*. *burgdorferi* FlaB (Affinity BioReagents) diluted 1:20,000. Appropriate anti-rabbit Ig or anti-mouse Ig HRP conjugates (Life Technologies) were diluted 1:5000 and used to detect primary antibodies on the PVDF membranes. Immune complexes were detected using the Western Lightning Chemiluminescent Reagent plus system (Perkin Elmer, Waltham, MA, USA).

### SPR

Direct binding of C1 and subunits of the C1 complex to BBK32 was assessed by SPR using a Biacore 3000 instrument (GE Healthcare) at 25°C. HBS-T-Ca^2+^ (20 mM HEPES (pH 7.3), 140 mM NaCl, 0.005% (v/v) Tween 20, 5 mM CaCl_2_) was used as the running buffer and a flowrate of 10 μl min^-1^ were used in all experiments. A BBK32 biosensor was created by immobilizing recombinant BBK32 proteins on separate flowcells of a C1 sensor chip (GE Healthcare). In all cases immobilization was achieved using standard amine coupling chemistry by activating the flowcell surface for 7 min at 5 μl min^-1^ with an equal volume mixture of 0.1 M N-hydroxysuccinimide and 0.4 M ethyl(dimethylaminopropyl) carbodiimide. Next, BBK32-FL at 20 μg ml^-1^ in 10 mM sodium acetate (pH 4.0), BBK32-N at 25 μg ml^-1^ in 10 mM sodium acetate (pH 4.5), or BBK32-C at 5 μg ml^-1^ in 10 mM sodium acetate (pH 5.5) were injected and allowed to react until the desired surface density was reached. Finally, 1 M ethanolamine (pH 8.5) was injected for 7 min at 5 μl min^-1^ to quench remaining reactive groups. A reference flowcell was generated by activating the surface followed by immediate quenching. Final immobilization densities reported in resonance units (RU) were as follows: BBK32-FL (165 RU), BBK32-N (150 RU), and BBK32-C (240 RU). All solution phase analytes were exchanged into running buffer just prior to injection.

Purified C1 was injected as a twofold concentration series in triplicate consisting of 0.20, 0.39, 0.78, 1.6, 3.1, 6.3, 13, 25, 50, and 100 nM for 3 min followed by 5 min of dissociation. The surface was regenerated to baseline by injecting HBS-T-EGTA (20 mM HEPES (pH 7.3), 140 mM NaCl, 0.005% Tween-20, and 10 mM EGTA) for 1 min followed by a 30 s injection of a solution containing 0.1 M glycine (pH 2.2) and 2.5 M NaCl. Direct interaction of C1r enzyme with BBK32 proteins was assessed using an identical protocol to C1 except for a concentration series consisting of 0.39, 0.78, 1.6, 3.1, 6.3, 13, 25, 50, 100, and 200 nM was used and the injection time was increased to 5 min followed by 10 min of dissociation. Binding of C1r enzyme to active BBK32 proteins was assessed on a second C1 biosensor with different coupling densities BBK32-FL (80 RU) and BBK32-C (330 RU). A separate concentration series consisting of 2.0, 3.9, 7.8, 16, 31, 63, 125, 250, 500, and 1000 nM was injected in duplicate for 5 min followed by 15 min of dissociation. Kinetic analysis was performed for each set of sensorgrams resulting from C1 or C1r enzyme injections using BIAevaluation software 4.1.1 (GE Healthcare) using a 1:1 (Langmuir) binding model and fitting R_max_ locally. To determine the effect of calcium on the interaction of BBK32, a single concentration of C1 (50 nM) or C1r enzyme (200 nM) was injected in triplicate in a running buffer of HBS-T-Ca^2+^ or HBS-T-EGTA. To evaluate relative binding of individual C1 complex components to BBK32, triplicate injections were performed at a fixed 50 nM concentration in HBS-T- Ca^2+^ running buffer. Response was corrected for the molecular weight of each component (C1 = 790 kDa, C1q = 410 kDa, C1r enzyme = 92 kDa, C1s proenzyme and C1s enzyme = 86 kDa).

### Complement inhibition ELISA

To delineate the effect of BBK32 proteins on the CP, LP, and AP, we adopted an ELISA based assay previously described [95]. Costar EIA/RIA plates (Fisher Scientific) were incubated overnight at room temperature with 3 μg ml^-1^ human IgM (CP initiator) (Athens Research & Technology), 25 μg ml^-1^ *Salmonella enteriditis* LPS (AP initiator) (Sigma Aldrich), or 20 μg ml^-1^ of mannan from *Saccharomyces cerevisiae* (LP initiator) (Sigma Aldrich) in a coating buffer consisting of 100 mM Na_2_CO_3_/NaHCO_3_ (pH 9.6). All subsequent steps were preceded by three consecutive washes with TBS-T buffer (50 mM Tris (pH 8.0), 150 mM NaCl, 0.05% (v/v) Triton X-100) and all reaction volumes were 100 μl. Plates were blocked in PBS-T-BSA (137 mM NaCl, 2.7 mM KCl, 10 mM Na_2_HPO_4_, 1.8 mM KH_2_PO_4_, 1% (w/v) bovine serum albumin, and 0.05% (v/v) Tween-20) for 1 h. Reactions consisted of pooled complement human serum (Innovative Research), at 1% (CP/LP) or 20% (AP) final concentration, various concentrations of BBK32 proteins, and CP/LP buffer (20 mM HEPES (pH 7.3), 0.1% (w/v) gelatin, 140 mM NaCl, 2 mM CaCl_2_, 0.5 mM MgCl_2_) or AP buffer (20 mM HEPES (pH 7.5), 0.1% (w/v) gelatin, 140 mM NaCl, 5 mM MgCl_2_, 10 mM EGTA). Serum/BBK32 mixtures were then added to wells and incubated at 37°C for 1 h. Downstream complement activation was measured by detecting C3b deposition using a 1:300 dilution of an anti-C3d monoclonal antibody (030–08, Santa Cruz Biotechnology) or 1:300 dilution of an anti-C4d monoclonal antibody (C4-1, Cell Sciences) incubated at room temperature for 1 h, and subsequent 1 h room temperature incubation with a 1:5000 dilution of goat anti-mouse HRP secondary antibody (Thermo Scientific). HRP-labeled antibody was detected using 1-step Ultra TMB (Thermo Scientific) for 10 min. The reaction was stopped by addition of 2 M sulfuric acid and the absorbance was measured at 450 nm using a VersaMax microplate reader (Molecular Devices). Wells containing serum only or where serum was replaced with buffer were treated as 100% and 0% signal, respectively. All experiments were performed a minimum of three times. IC_50_ values were evaluated by variable slope four-parameter nonlinear regression analysis performed using GraphPad Prism 5.0.

### Hemolytic assays

Inhibition of CP-mediated hemolysis by BBK32 proteins was assessed using a modified classical pathway hemolytic assay (CP50). Sheep erythrocytes (5 x 10^8^ cells ml^-1^) sensitized with human IgM (Complement Tech) were centrifuged at 500 x *g* at 4°C for 3 min and resuspended in GHB^++^ buffer (20 mM HEPES (pH 7.3), 140 mM NaCl, 0.1% gelatin (w/v), 0.15 mM CaCl_2_, and 0.5 mM MgCl_2_). Final volumes were 100 μl and reactions began by mixing 35 μl GHB^++^ with 20 μl of BBK32 proteins previously diluted into GHB^++^ at various concentrations, followed by 20 μl NHS (1% v/v) final concentration, and 25 μl sheep erythrocytes sensitized with human IgM. Reactions were incubated at 37°C for 1 h with intermittent shaking and clarified by centrifugation at 1000 x *g* for 3 min. 50 μl of each reaction were transferred to a 96-well flat-bottom half-area microplate and absorbance was measured at 541 nm using a VersaMax microplate reader (Molecular Devices). A well containing no BBK32 was considered as 100% lysis and background absorbance was measured by replacing NHS with buffer. Percent lysis was calculated by subtracting background readings from each well and comparing each reading to 100% controls.

Inhibition of AP-mediated hemolysis by BBK32 proteins was assessed using a modified alternative pathway hemolytic assay (APH50). Rabbit erythrocytes (Complement Tech) at 5 x 10^8^ cells ml^-1^ were washed by centrifugation at 500 x *g* at 4°C for 3 min and resuspension in GHBS° buffer (20 mM HEPES (pH 7.5), 140 mM NaCl and 0.1% gelatin (w/v)). Reactions began by diluting 5 μl of 0.1M MgCl_2_-EGTA into 30 μl GHBS°, followed by 20 μl of BBK32 proteins, followed by 20 μl of NHS, and finally 25 μl rabbit erythrocytes. Reactions were allowed to incubate at 37°C for 30 min with intermittent agitation, clarified, and diluted 1:10 in a 96-well plate. Absorbance was measured at 412 nm and % lysis was computed as described for the CP50 assay. All experiments were repeated between two and four times. IC_50_ values were evaluated by variable slope four-parameter nonlinear regression analysis performed using GraphPad Prism 5.0.

### C1r/C1s enzyme activity assays

The ability of BBK32 proteins to inhibit the enzymatic cleavage of C1s proenzyme by C1r enzyme *in vitro* was performed as follows. Reactions were carried out in HBS-Ca^2+^ (20 mM HEPES (pH 7.3), 140 mM NaCl, 5 mM CaCl_2_). Reaction volumes were 10 μl and consisted of 5 μl BBK32 protein previously diluted into water, 1 μl C1s proenzyme (1 μg μL^-1^), 1.5 μl C1r enzyme (333 nM), and 2.5 μl of 4x HBS-Ca^2+^. Following overnight incubation at 37°C, each reaction was mixed with 5 μl reducing SDS-PAGE Laemmli sample buffer, boiled for 5 min, and 7.5 μl of each reaction were separated on a 10% Tris-tricine SDS-PAGE gel. Following Coomassie blue staining, digital images of destained gels were captured using a FluorChem M imaging system (ProteinSimple). Densitometry was performed using AlphaView SA 3.4.0 software (ProteinSimple) and the normalized peak height of the band corresponding to C1s proenzyme was plotted against the concentration of BBK32 present in each reaction. IC_50_ values were evaluated with GraphPad Prism 5.0 using four parameter variable slope nonlinear regression analysis and constraining the top and bottom values to 100 and 0 respectively. All experiments were repeated between two and four times.

The ability of BBK32 proteins to inhibit the enzymatic cleavage of C4 by C1s enzyme *in vitro* was performed as follows. Reaction volumes were 10 μl and consisted of 2.5 μl C4 (1 mg ml^-1^), 1 μl C1s enzyme (1 mg ml^-1^), 1.5 μl PBS, and 5 μl of BBK32 proteins previously diluted into PBS. Reactions were incubated at 37°C for 30 min and stopped by the addition of 5 μl SDS-PAGE reducing buffer, boiled for 5 min, and evaluated by SDS-PAGE.

### C1q co-immunoprecipitation

Monoclonal antibodies to human C1q were captured by Protein G beads (Thermo Scientific) at room-temperature for 2 h as instructed by the manufacturer. Purified C1 complex (40 nM) was incubated at room temperature for 2 h with either 5 μM BBK32-FL, BBK32-N, BBK32-C, or full-length recombinant OspC. All reactions were performed in HEPES^++^ buffer (20 mM HEPES, 140 mM NaCl, 0.15 mM CaCl_2_ and 0.5 mM MgCl_2_, pH 7.3). Subsequently, C1, pre-incubated with borrelial recombinant proteins, was added to Protein G beads pre-loaded with C1q monoclonal antibodies and incubated at 37°C for 2 hr. The beads were then washed 5 times with HEPES^++^ buffer containing 0.2% Tween-20, followed by the addition of 50 μl Laemmli sample buffer. Samples were then boiled for 10 min, separated by SDS-PAGE, transferred to PVDF membranes, and subjected to Western immunoblot analysis. The components of the C1 complex or BBK32 were detected with polyclonal goat anti-human C1q (diluted 1:5000), goat anti-human C1r (diluted 1:3000), sheep anti-human C1s (diluted 1:3000), or rabbit polyclonal antibody to BBK32 (diluted 1:1500) followed by incubation with either HRP-conjugated TrueBlot (Rockland Antibodies) directed against goat or sheep immunoglobulin (diluted 1:2000) (for the C1 proteins) or goat anti-rabbit Ig/HRP (Life Technologies) diluted 1:5000 (for BBK32). The membranes were washed extensively in PBS, 0.2% Tween-20, and developed using the Western Lightning Chemiluminescent Reagent plus system (Perkin Elmer, Waltham, MA, USA).

### *B*. *burgdorferi* whole cell adherence assays

*B*. *burgdorferi* adherence assay was done as previously described with slight modifications [94]. Briefly, poly-D-lysine pre-coated coverslips (Corning Biocoat) were coated with 1 μg human C1 (Comptech), C1r (Comptech), or BSA respectively and incubated at 4°C overnight. The coverslips were washed thoroughly in PBS to remove excess unbound proteins. The coverslips were then blocked with 3% BSA at room temperature for 1 hr. *B*. *burgdorferi* strains ML23/pBBE22*luc* and JS315/pBBE22*luc* were grown to mid-logarithmic phase at 37°C, 5% CO_2_, pH 6.8 to induce expression of *bbk32*. Strains B314/pBBE22*luc* and B314/pCD100 were grown to mid-logarithmic phase at 32°C, 1% CO_2_, pH 7.6 since the production of BBK32 in B314/pCD100 was high independent of growth condition. All *B*. *burgdorferi* strains were subsequently diluted to 10^7^ organisms/ml in BSK-II medium without serum. The resulting *B*. *burgdorferi* samples, in 0.1 ml volumes, were applied onto the coverslips and incubated for 2 hr at 32°C. Unbound bacteria were removed from the coverslips by gentle washing with PBS; this wash step was repeated 7 times. The coverslips were applied to a glass slide and attached spirochetes were counted by dark field microscopy. Binding of spirochetes to their respective targets was scored by dark field microscopy.

### Serum complement sensitivity assay

Complement sensitivity assays were performed as previously described [27,96]. Briefly, *B*. *burgdorferi strains* were grown to exponential phase at 32°C, 1% CO2, pH 7.6, and 10^6^ cells suspension in 80 μl of BSK-II medium was added to 20 μl of normal human serum (NHS) or 10 mM Mg/EGTA treated NHS to give a final volume of 100 μl with 20% serum. The samples were placed in microtiter plates and the suspensions were sealed and incubated at 32°C for 2 h. Heat-inactivated normal human serum (hiNHS) was used as a control. After incubation, *B*. *burgdorferi* suspensions were scored under dark field microscope and the percentage of viable *B*. *burgdorferi* cells were calculated from randomly chosen fields and based on immobilization, loss of cell envelope integrity, and overt lysis.

## Supporting Information

S1 Fig The interaction of BBK32 with C1r is calcium dependent. As was observed for the C1 complex, the interaction of C1r with (A) full-length BBK32-FL or (B) BBK32-C is strongly dependent on calcium as judged by SPR.
(TIFF)

S2 Fig Infectious *B*. *burgdorferi* and the isogenic *bbk32* mutant bind to C1 at high levels. Strain ML23/pBBE22*luc* and the *bbk32* mutant derivative JS315/ pBBE22*luc* were incubated with immobilized C1 to assess binding. No significant difference was observed in binding for both strains tested. Each data point represents spirochetes counted within an independent field as scored by dark field microscopy (n = 30 per strain). The horizontal bar represents the mean value. Statistical significance was evaluated by use of an unpaired *t* test. ns; not significant.
(TIFF)
