# Supplementary figures and images for: Borrelia burgdorferi BBK32 Inhibits the Classical Pathway by Blocking Activation of the C1 Complement Complex
Source: PLoS Pathog. 2016 Jan 25;12(1):e1005404. doi: 10.1371/journal.ppat.1005404 (PMC4725857; doi:10.1371/journal.ppat.1005404)

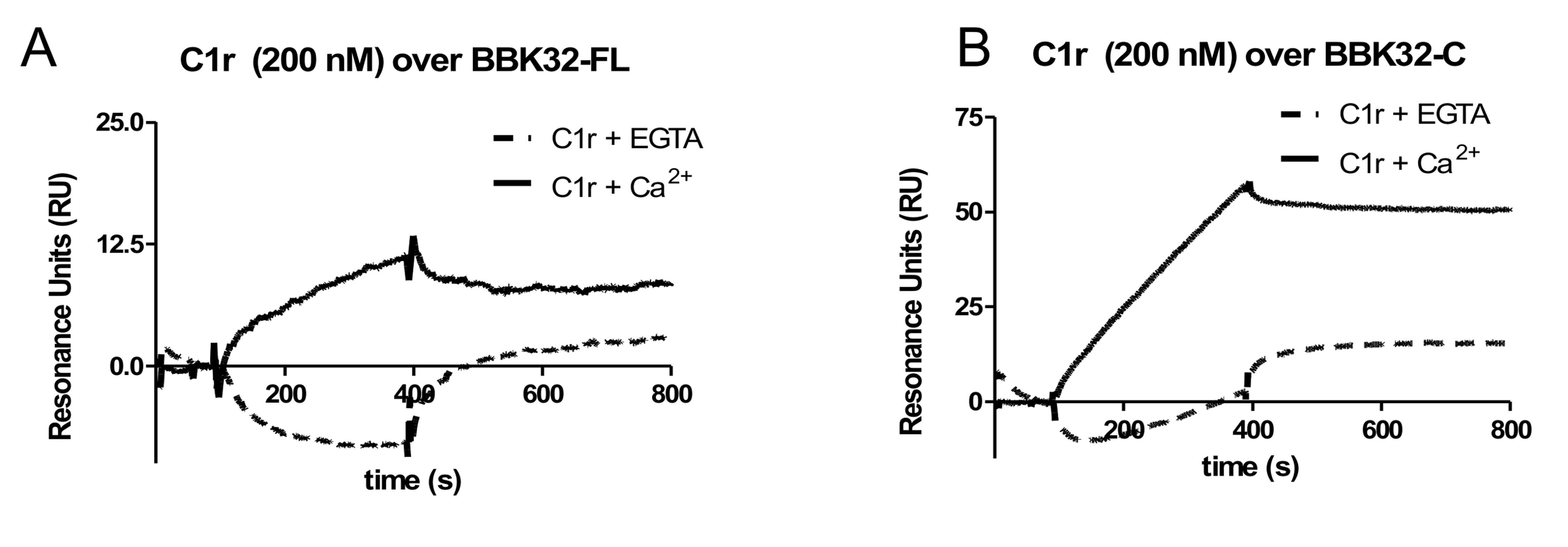

Supplement: S1 Fig — As was observed for the C1 complex, the interaction of C1r with (A) full-length BBK32-FL or (B) BBK32-C is strongly dependent on calcium as judged by SPR. (TIFF) [file ppat.1005404.s001.tiff]

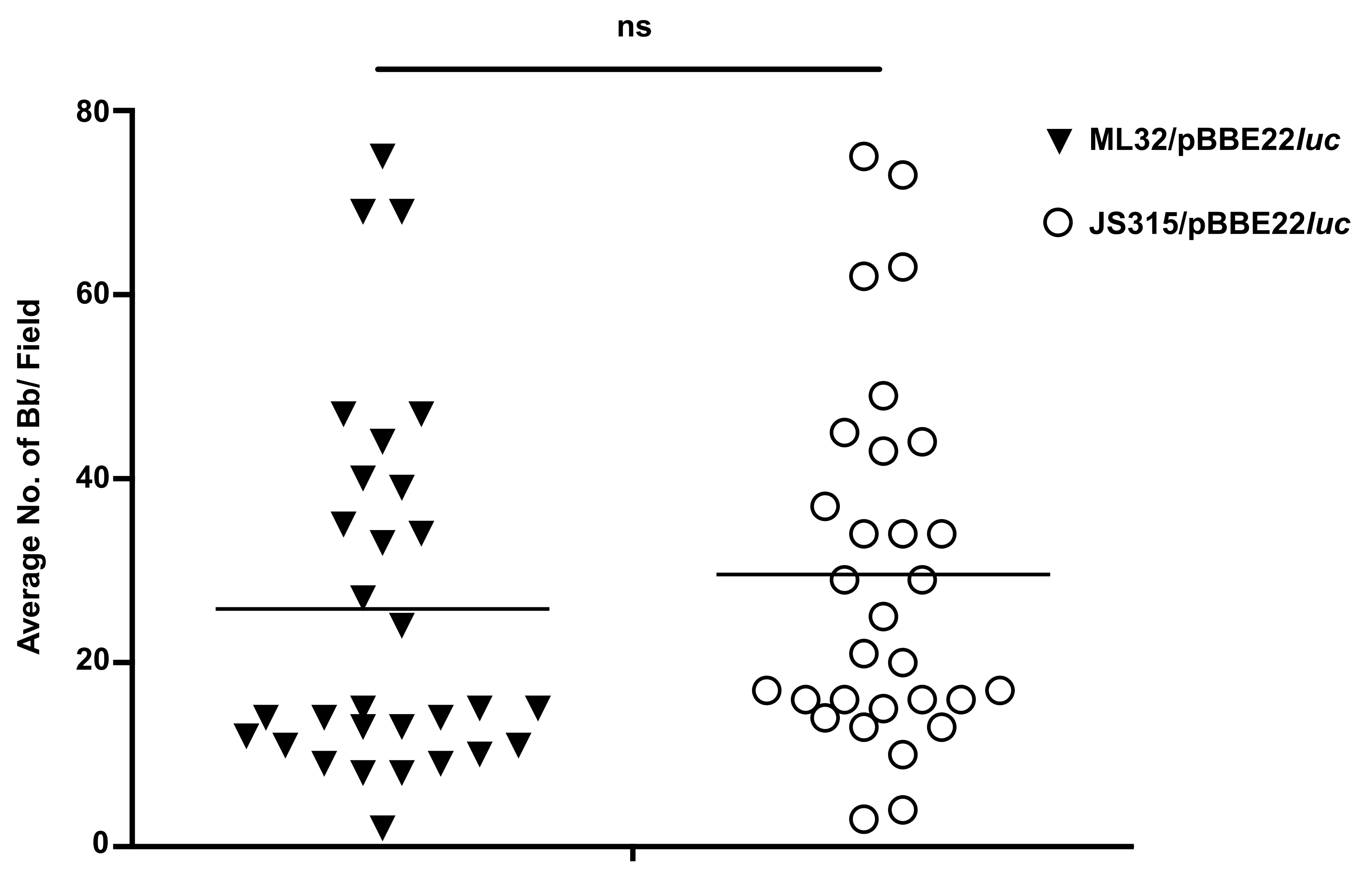

Supplement: S2 Fig — Strain ML23/pBBE22luc and the bbk32 mutant derivative JS315/ pBBE22luc were incubated with immobilized C1 to assess binding. No significant difference was observed in binding for both strains tested. Each data point represents spirochetes counted within an independent field as scored by dark field microscopy (n = 30 per strain). The horizontal bar represents the mean value. Statistical significance was evaluated by use of an unpaired t test. ns; not significant. (TIFF) [file ppat.1005404.s002.tiff]
